# High-throughput detection of microeukaryotic parasites in insects using Nanopore sequencing

**DOI:** 10.1099/mgen.0.001649

**Published:** 2026-03-16

**Authors:** Edouard Bessette, Sam Edwards, Bryony A. P. Williams

**Affiliations:** 1 Department of Biosciences, Faculty of Health and Life Sciences, University of Exeter, Exeter, EX4 4QD, UK; 2 Department of Plant and Environmental Sciences, University of Copenhagen, Thorvaldsensvej 40, 1871 Frederiksberg C, Denmark

**Keywords:** Nanopore MinION, microeukaryotes, parasites, reared insects, phylogenetics, pathogen surveillance

## Abstract

The mass rearing of insects for food and feed is increasing significantly, but it presents challenges in detecting and characterizing pathogens which may cause severe economic losses. Much attention is placed on detecting viruses, bacteria and fungi; however, other significant entomopathogens remain overlooked, such as protist and microsporidian parasites. This study employed Nanopore sequencing to explore and phylogenetically analyse microsporidian and protist parasites in reared insects. Using group-specific primers and amplicon pooling strategies, we successfully amplified and sequenced a key protist group (Gregarinasina), microsporidia and fungal pathogens from insect host DNA, providing data for their future detection. Our approach generated novel molecular data, enabling the inference of small subunit and long subunit rRNA phylogenies which enhance the understanding of the evolutionary origin of these parasites. Furthermore, we highlight potential shortcomings of currently available primers and provide new Gregarine-specific primers to aid in further research of these overlooked entomopathogens. This research demonstrates the effectiveness of third-generation sequencing technologies in improving diagnostic tools and advances the study of pathogen biodiversity within economically important insect hosts.

**Impact Statement**

This study introduces an efficient, high-throughput method for detecting diverse microeukaryotic parasites in insect hosts using pooled amplicon Nanopore sequencing. Our approach enables sensitive pathogen identification from minimal DNA and reveals previously unknown parasite diversity. The validated primers and bioinformatics pipeline can support routine, cost-effective pathogen screening in insect farming, helping to safeguard production and advance food security.

## Data Summary

This study generated Nanopore sequencing data from amplicon libraries targeting protist and microsporidian parasites in insect hosts. All raw sequencing data, consensus 18S/28S sequences, sample table (Samples_Seq_table.xlsx) and bioinformatics scripts are publicly available either through GitHub (Amplicon-high-throughput-ONT-sequencing) or Zenodo repository (DOI: 10.5281/zenodo.15129942). All supporting phylogenetic alignments and raw data files used for tree construction can be accessed through the Phylogeny-Alignments repository link (Phylogeny-Alignments).

## Data Availability

Our research contributes to reproducibility and transparency by making all raw data and analysis scripts publicly available through a GitHub (Amplicon-high-throughput-ONT sequencing: ONT sequencing of protists and microsporidia 18S/28S amplicons) and a Zenodo repository (Zenodo.15129942).

## Introduction

Insect mass rearing for food and feed requires robust diagnostic tools to detect pathogens beyond well-studied bacteria, viruses and fungi. Protist and microsporidian parasites can thrive in high-density conditions, causing severe economic losses and colony collapse [1,2]. For example, the amoeba *Malamoeba locustae* is known to infect reared orthopterans, causing premature death [3,4]. Similarly, the gregarine *Gregarina cuneata* affects the mealworm *Tenebrio molitor*, leading to higher mortality, weight loss and developmental delays [5,6]. Microsporidian parasites are known to induce a general reduction in host fitness across multiple insect hosts, resulting in higher mortality, reduced longevity, weight loss and developmental delays [2,7].

Despite their impact, many entomopathogenic protists lack molecular data for diagnostics [8]. Although metabarcoding has emerged as a powerful approach for assessing microbial diversity, these pathogens are often overlooked. Comprehensive molecular data collection could improve our understanding of their phylogenetic relationships, particularly in neglected clades such as Gregarinasina or Amoebozoa [8]. However, conventional metabarcoding approaches typically rely on short-read sequences, which restrict the phylogenetic resolution and can prevent accurate species delineation. Third-generation sequencing (TGS) allows the sequencing of longer fragments, improving taxonomic identification and phylogenetic accuracy [9,10]. A current challenge for TGS compared to Illumina (second-generation sequencing) is the variable sequencing error rates, ranging from 1 to 15%, depending on the used platforms [11,14]. This challenge has been addressed by the creation of novel tools and approaches that can perform *de novo* clustering of long noisy ONT reads into operational taxonomic units, such as NGSpeciesID, reaching more than 99% accuracy [12,15,17]. Until now, DNA barcoding analyses have predominantly relied on QIIME2, which is widely used for 16S amplicons [18,19]. However, QIIME2’s support for long-read amplicon analysis remains limited [12].

In this study, we used short- and long-read metabarcoding and cost-effective MinION sequencing to generate molecular data for poorly characterized protists and improve diagnostics. Targeting entomopathogenic protists and Microsporidia, we employed group-specific primers, as no universal protist primers exist due to host-parasite phylogenetic overlap [20]. Anti-metazoan primers [21] and a reverse large subunit (LSU) rRNA gene primer [9] enhanced detection and phylogenetic resolution.

A key strength of this study was pooling amplicons from different primer sets before sequencing, a strategy previously explored for mitochondrial and nuclear genes [22] and aquatic microbial communities [23]. This method improves efficiency compared to metagenomics while enabling accurate recovery of taxonomic markers using tools like Minibar and cutadapt [24,25]. Using this approach, we identified protists and other pathogens in reared insects and generated novel genomic data for microsporidia, gregarines and fungi. Moreover, small subunit (SSU) rRNA gene and LSU phylogenies provided new insights into protist biodiversity. By integrating TGS with amplicon pooling, this study refines long-read metabarcoding and enhances diagnostic tools for parasites in insects of economic importance.

## Methods

### Insect samples

Host insects investigated in this study were either commercially reared or wild-caught. Reared insects were purchased in four different pet shops: Exeter Exotics (Exeter, UK), Pets at Home (Exeter, UK), Live Foods (UK) and Monis (Copenhagen, Denmark). The following reared insect species were screened for microsporidia and protist parasites: *Zophobas morio*, *T. molitor*, *Schistocerca gregaria*, *Gryllus bimaculatus*, *Gryllus assimilis* and *Galleria mellonella*. In different locations around Exeter, UK, the following selected wild Lepidoptera were sampled and screened: *G. mellonella* from Streatham Campus, University of Exeter (50° 44′ 14.0″ N 3° 32′ 03.5″ W) and Cockington apiary, Torbay (50° 27′ 49.7″ N 3° 33′ 50.5″ W), where larvae or adults were collected from active beehives; *Aphomia sociella* from Streatham Campus, University of Exeter, collected from bumblebee colonies; and *Pieris rapae* from Shillingford Organics, Exeter (50° 41′ 14.4″ N 3° 33′ 04.4″ W), where caterpillars were hand-collected and adults were caught with butterfly nets from cabbage crops.

### Insect dissection and DNA extraction

All reared and wild insects were processed using the same protocol. Insects were anesthetized by freezing before dissection. Under a stereomicroscope, the gut and fat tissues of an individual insect were extracted in a Petri dish and triturated with fine forceps in ca. 250 µl of 1× PBS. Although gregarines were occasionally observed as large parasites during tissue dissection, microscopic detection of parasites was not a prerequisite for sample processing; all dissected insects were subjected to DNA extraction regardless of visible infection signs. The formed ‘dissection pool’ and tissues were collected in a 2-ml SafeLock tube for later DNA extraction. The DNA was extracted using a Cetyltrimethylammonium bromide (CTAB) procedure including phenol/chloroform/isoamyl. Briefly, tissues were homogenized with a FastPrep Cell Disrupter (Qbiogene, Carlsbad, CA) in a mix of 500 µl CTAB buffer and 400 µl glass beads (0.17–0.18 mm Ø, Sigma-Aldrich). The mixture was then incubated with 10 µl of proteinase K (400 µg ml^−1^) at 54 °C overnight. After cooling, 2 µl of RNaseA (10 µg ml^−1^) was added, and the solution was incubated at 37 °C for 20 min. Thereafter, the genomic DNA was extracted in 700 µl of phenol/chloroform/isoamyl alcohol (25 : 24 : 1) followed by a single extraction in chloroform [26]. The DNA was finally precipitated with 3 M NaOAc (0.1× the sample volume) and absolute ethanol (2.5× the total volume). The DNA was eventually dissolved in 50 µl of sterile water and stored at −20 °C for later PCR. A total of 296 DNA samples were extracted and used for further analysis. Sample information can be accessed in the following: Samples_Seq_table.xlsx.

### Gregarine DNA extraction and primer design

Gregarines were occasionally observed during dissection, and individual gregarines from *Acheta domesticus* and *Alphitobius diaperinus* were isolated for DNA extraction. Gregarines were gently spread out by triturating the insect host gut tissues with fine forceps in 1× PBS and transferred with a 200 µl pipette (avoiding gut tissue remnants) to a new glass Petri dish with fresh PBS for washing. The gregarine cells were then transferred to a 1.5-ml Eppendorf tube, minimizing PBS volume, and centrifuged for 30 s in a microcentrifuge. The pelleted gregarine cells were then used for CTAB genomic extraction as described in Insect dissection and DNA extraction. The samples were identified as ‘T Ga’ and ‘T Lg’ (Samples_Seq_table.xlsx). The obtained pure gregarine DNA was used to validate the designed primers, as a positive control, and provide reliable genomic data for subsequent phylogenetic analysis (unpublished data).

Gregarines are an important protist group infecting reared insects, yet no primer set was available for this group in the PR2 primer database v2 [27], so new 18S primers were designed based on Gregarinoidea (Terrestrial Gregarine clade) sequences from GenBank. The forward primer targeted a conserved region (approximate positions 1,200–1,400 bp) in the reference sequences, as indicated by a low mean entropy (0.11±0.05). By contrast, the reverse primer targeted a more variable region (~1,500–1,700 bp) with the aim of retrieving a more diverse species set, as shown by a higher mean entropy (0.74±0.05). The primers were designed to amplify a short region of ca. 350 bp. Primer specificity was tested in *silico* using TestPrime (silva) (Table S1). The alignment of the gregarine reference sequences used for primer design and the corresponding primer set is available in the following: Gregarine-Primer-design.

### DNA pooling and PCR

DNA extracts were pooled by host species and used for PCR with universal and group-specific primers targeting protists and microsporidia. Group-specific primers were combined with the broad LSU primer (iLSUr) for long amplicons (Table 1, Fig. S1, available in the online Supplementary Material). Their purity was assessed using a NanoDrop™ Lite Spectrophotometer (Thermo Fisher Scientific), and their concentrations were determined with a Qubit 4™ Fluorometer (Thermo Fisher Scientific) (Fig. S2). When assessing the DNA concentration of the initial samples, if it was assessed ‘too low’ by Qubit, or below 10 ng µl^−1^, the extract was concentrated by ethanol precipitation and elution in a smaller volume of water (10 µl). To form the host DNA pools, each DNA sample was diluted to 10 ng µl^−1^ in a 10 µl solution (v/v, Milli-Q water), and 2 µl from each sample was pooled (i.e. 20 ng of DNA), yielding a final volume dependent on the number of individuals per species.

**Table 1.** List of primer pairs used to perform the PCRs Tm: Melting or annealing temperature used during the PCR programme with Phusion polymerase (see the different used programmes in File S1). When the reverse primer iLSUr [77] is present, it means that positive samples were found within the targeted group and further used to produce long amplicons.

Parasite group	Primer name	Sequence (5′−3′)	Product size (bp)	Tm (°C)	Primer reference
**Microsporidia**	V1F/CM-V5R	F: CACCAGGTTGATTCTGCCTGAC R: TAANCAGCACAMTCCACTC	ca. 780	59	[77,81]
	V1F/530R	F: CACCAGGTTGATTCTGCCTGAC R: CCGCGGCKGCTGGCAC	ca. 400	69	
	V1F/iLSUr	F: CACCAGGTTGATTCTGCCTGAC R: CCGCGGCKGCTGGCAC	ca. 3,500	67	
	CM-V5F/iLSUr	F: GATTAGANACCNNNGTAGTTC R: ACCTGTCTCACGACGGTCTAAAC	2,800–3,000	55	
	CM-V5R(rc)/iLSUr	F: GAGTGGAKTGTGCTGNTTA R: ACCTGTCTCACGACGGTCTAAAC	2,700–2,900	58	
**Gregarinasina**	WL1/EukP3	F: GCGCTACCTGGTTGATCCTGCC R: GACGGGCGGTGTGTAC	ca. 1,600	55	[82]
	1-18S-GRF/1222-18S-GRR	F: CGGTAATTCCAGCTCCAAT R: TGACTTGCGCTTACTAGGG	ca. 950	55	[83]
	GregF/GregR	F: CCCTTAGATRRYCTGGGCTGC R: CGTGTTACGACTTCTTC	ca. 330	55	Designed; [77]
	GregF/iLSUr	F: CCCTTAGATRRYCTGGGCTGC R: ACCTGTCTCACGACGGTCTAAAC	3,300–4,000	64	
	GregR_RC/iLSUr	F: GAAGAAGTCGTAACACG R: ACCTGTCTCACGACGGTCTAAAC	3,000–3,700	56	
**Cryptosporidia**	SCL2/SCR2	F: CAGTTATAGTTTACTTGATAATC R: CAATACCCTACCGTCTAAAG	200	58	[84]
**Chlorophyta**	MGF/MGR	F: AGGATAGAGGCCTACCATGGTTTCAA R: CCCCGACTGTCCCTCTCCAT	569	67	[85]
**Amoebozoa**	AmoebozoaF/AmoebozoaR	F: GAATTGACGGAAGGGCACAC R: CCAAGAYRTCTAAGGGCATCAC	360–420	60	[77,86]
	AmoebozoaF/iLSUr	F: GAATTGACGGAAGGGCACAC R: ACCTGTCTCACGACGGTCTAAAC	ca. 4,500	66	
	AmoebozoaR(rc)/iLSUr	F: GTGATGCCCTTAGAYRTCTTGG R: ACCTGTCTCACGACGGTCTAAAC	ca. 4,200	64	
**Ichthyosporea**	500F/900R	F: CGGCTACCACTTCTACGGAGG R: ATTAACGCCCCCAACTATCCC	470–509	66	[87]
**Apicomplexa**	Apicomplexa B F/Apicomplexa B R	F: TGYGTTTGAATACTAYAGCATGG R: TCTGATCGTCTTCACTCCCTT	228–244	62	[86]
**Kinetoplastea**	Kineto14F/kineto2026R	F: CTGCCAGTAGTCATATGCTTGTTTCAAGGA R: GATCCTTCTGCAGGTTCACCTACAGCT	1,900–2,200	68	[77,88]
	Kineto14F/iLSUr	F: CTGCCAGTAGTCATATGCTTGTTTCAAGGA R: ACCTGTCTCACGACGGTCTAAAC	6,000–7,000	67	
	kineto2026R(rc)/iLSUr	F: AGCTGTAGGTGAACCTGCAGAAGGATC R: ACCTGTCTCACGACGGTCTAAAC	ca. 5,000	67	
**Universal non-metazoan primers**	574*f/UNonMet_DB	F: CGGTAAYTCCAGCTCYV R: CTTTAARTTTCASYCTTGCG	ca. 574 (median length)	56	[21,89]

Initial ‘short’ PCRs amplified SSU regions using universal and group-specific primers to identify positive samples. These samples were then subjected to a second ‘long’ PCR targeting both SSU and LSU regions, using the same forward 18S primer with the iLSUr reverse primer. Short and long PCR products were purified (Qiagen Gel PCR Clean-up). If the gel showed only the expected band, the entire mixture was purified; otherwise, the correct band was excised and purified. Nested PCRs were performed using long PCR amplicons as templates. The reverse complement of the group-specific reverse primer previously used served as the new forward primer, paired with iLSUr, to improve amplification.

Each 20 µl PCR reaction contained 2 µl of DNA (~20 ng), 1 µM primers, 200 µM dNTPs, 1× Phusion HF Buffer and 0.02 U Phusion DNA polymerase (NEB). The thermocycler programme included an initial 98 °C denaturation (30 s), 30 cycles of 98 °C denaturation (10 s), annealing (30 s) and 72 °C extension (2 min), with a final 72 °C extension (10 min). Long and nested PCRs used similar conditions with a 2 min 30 s extension. Negative PCR reactions were always undertaken with water used as template and optimal annealing temperature for each primer used.

PCR products (5 µl) were run on a 1.3% agarose gel (pre-mixed with SafeView™ dye) at 100 V for 30 min in TAE buffer and visualized under UV. Expected bands were excised, purified (Qiagen Gel PCR Clean-up), pooled by insect species and quantified (Qubit dsDNA HS Assay Kit, Thermo Fisher).

All the performed PCRs and respective gels can be found in the supplementary file 1 ‘Laboratory notes and PCR gels’ (Supplementary Material).

### Oxford Nanopore sequencing

Nanopore sequencing followed the ONT native barcoding protocol vNBA_9093_v109_revJ_12Nov2019 using EXP-NBD104 and SQK-LSK109 kits [28]. Library preparation involved native barcode and sequencing adapter ligation. The final library, prepared as recommended, was purified (AMPure XP beads), quantified (Qubit) and sequenced on a MinION (Oxford Nanopore Technologies) using an R9 flow cell. ONT MinKNOW software collected raw sequencing data in fasta5 format. Three MinION runs were performed (Table S2).

### Bioinformatic analysis

Raw fast5 reads were basecalled using Guppy v6.4.8 (*guppy_basecaller*) with a minimum quality score of 7 and the *dna_r9.4.1_450bps_hac.cfg* configuration. Reads were then demultiplexed (*guppy_barcoder*) with *--detect_mid_strand_barcodes* to improve barcode detection. Fastq files were merged and processed with an in-house bash script.

Briefly, reads were trimmed using cutadapt v3.5 [24], with the non-metazoan and group-specific primers for short SSU amplicons and the reverse iLSUr for long amplicons (Table 1). For long reads, only the nested primer was searched to avoid repetitive sequences. A 0.1 *error rate* was applied for primer alignment [29]. Quality filtering was performed using fastq_quality_trimmer (FASTX Toolkit v0.0.14) [30] and seqkit [31] based on the expected amplicon size. Consensus sequences were generated with NGSpeciesID [17], using the options *ont*, *consensus*, *medaka* and *sample size* 300, as recommended for ONT amplicon sequencing data [25,32, 33]. If more than one consensus was formed, due to co-amplifications, only the consensuses classified as an expected taxon (see below) were further kept for phylogenetic analysis [22] [Amplicons Table, see the column ‘Included in phylo. Analysis’].

The general reproducible bash script is available via the following link, amplicon.sh, and can be adapted with custom primers and expected amplicon length. Demultiplexed ONT fastq reads, cutadapt intermediate files, NGSpeciesID outputs and specific scripts are accessible at Zenodo.15129942.

Amplicon consensus sequences were assigned taxonomically via megablast against the NCBI nt database, using the hit with the highest bit score to select the best taxonomic match. We did not apply a fixed sequence identity cutoff, as our aim was to retain the closest available matches. All megablast searches were run online against the NCBI nt database [34] accessed in November 2024. Validated consensuses that showed an expected taxon and had more than one supporting read were gathered by group (Gregarinasina, Microsporidia and Fungi) and clustered with CD-HIT [35] at a 98% threshold, commonly used for species delimitation with 18S markers [36,39], before phylogenetic analysis.

The specificity of each primer set was eventually assessed by dividing the number of consensus sequences corresponding to the expected target taxon by the total number of recovered consensus sequences:


$$\text{Specificity (\textbackslash{}\%)}=\frac{\text{Number of expected taxon consensuses}}{\text{Total recovered consensuses}}\times 100$$

### Phylogenetic analysis

Standard blastn search (*E*-value cutoff of 1e-10) was used to identify homologous sequences for each Nanopore amplicon consensus against the NCBI non-redundant database [40,41]. The top hits (lowest e-value) were downloaded and aligned with the amplicon consensus sequences using MAFFT v7 [42] and then trimmed with BMGE -g 0.3 [43]. Phylogenies for the different recovered taxa were constructed separately for the SSU and LSU genes, when possible. Maximum-likelihood trees were built with FastTree v2.1.11 and the general time reversible (GTR) model (gamma option, 1,000 bootstraps) [44,45]. Trees were visualized with the *ape* and *ggtree* R packages and eventually edited in Adobe Illustrator [46,48].

One concatenated tree was constructed by creating a matrix from gregarine consensuses recovered from the same host that had both 18S and 28S data. For this, trimmed and curated sequences were concatenated with FASconCAT-G [49], and the tree was made with the same method as above. For the gregarine–host co-phylogeny, one representative nucleotide sequence of the cytochrome c oxidase subunit I (COI) gene for each host species was downloaded from GenBank. A maximum-likelihood tree with 1,000 bootstraps was constructed as above using MAFFT and BMGE, with the exception of using iqtree2 and the GTR+G5+I model [50,53]. The different raw files used for the phylogenetic analyses can be found in the following: Phylogeny-Alignments.

## Results

### Sequencing output and primer performance

Three Nanopore MinION runs generated a total of 1,995,585 reads after basecalling and demultiplexing. Nanopore barcode scores ranged from 82.5 to 96.3, indicating successful ONT barcode assignment and ligation (Table S1). The ‘Run 2’ showed substantially lower read counts due to low ONT library concentration. However, this run still yielded sufficient coverage for consensus generation.

The newly designed GregF/GregR primer set, targeting Gregarinasina, generated 45,758 reads [as determined by cutadapt; see the Amplicons Table]. These reads produced 15 gregarine consensus sequences (each supported by at least two ONT reads) using NGSpeciesID and blast search, yielding a gregarine specificity of 75% (i.e. the number of expected taxa divided by all taxa recovered from NGSpeciesID consensuses). By contrast, the 1-18S-GRF/1222-18S-GRR primer set generated 18,965 reads but did not produce any gregarine consensuses, indicating no gregarine specificity (Table 2). When used with a broad LSU reverse primer, the nested PCR primer set GregRC/iLSUr, where GregRC (the reverse complement of GregR) is applied as a nested primer following a long PCR with GregF and iLSUr, generated 54 reads and produced 11 gregarine consensus sequences. The AmoebozoaF/AmoebozoaR pair, expected to amplify amoebas, produced 2,182 reads but only generated insect consensus sequences, indicating low specificity (Table 2). For Microsporidia, the V1F/530R set generated 18,824 reads and allowed to recover 6 microsporidia consensus sequences, showing high specificity (Table 2). The non-metazoan 574*f/UNonMet_DB primer set retrieved 1,809 reads from which 3 gregarine and 9 fungal consensuses were created (Table 2).

**Table 2.** Different primer pairs used and the taxonomy assignment of the obtained amplicon consensuses (results shown for consensuses supported by at least two reads) The taxonomy was determined via blastn (NCBI nt database). Primer reads correspond to the number of reads successfully recovered and trimmed by cutadapt [24]. The specificity was determined, for a given primer pair, by dividing the number of expected consensuses by the total of produced consensuses. Data used to construct this table are available in the following: Amplicons Table. Greg, gregarine; Microsp., microsporidia.

Targeted group	Primer set	Primer reads	NGSpeciesID consensus							Specificity (%)
			Fungi	Fungi *incertae sedis*	Greg.	Microsp.	Bacteria	Virus	Insect	
Gregarinasina	1-18S-GRF/1222-18S-GRR	18,965	5	-	-	1	-	-	2	0
	WL1/EukP3	289	-	-	1	-	-	-	1	50
	GregF/GregR	45,758	-	-	15	-	3	1	1	75
	GregR_RC/iLSUr	54	-	-	11	-	-	-	-	100
Amoebozoa	AmoebozoaF/AmoebozoaR	2,182	-	-	-	-	-	-	15	0
	AmoebozoaR_RC/iLSUr	3	2	-	-	-	-	-	-	0
Kinetoplastea	Kineto14F/kineto2026R	72	9	1	4	-	-	-	-	0
	Kineto2026R_RC/iLSUr	12	-	-	4	-	-	-	-	0
Microsporidia	V1F/530R	18,824	-	-	1	6	1	-	-	75
Non-metazoa	574*f/UNonMet_DB	1,809	7	2	3	-	-	-	1	92

## Phylogenetic results

### Microsporidia

**Fig. 1.**
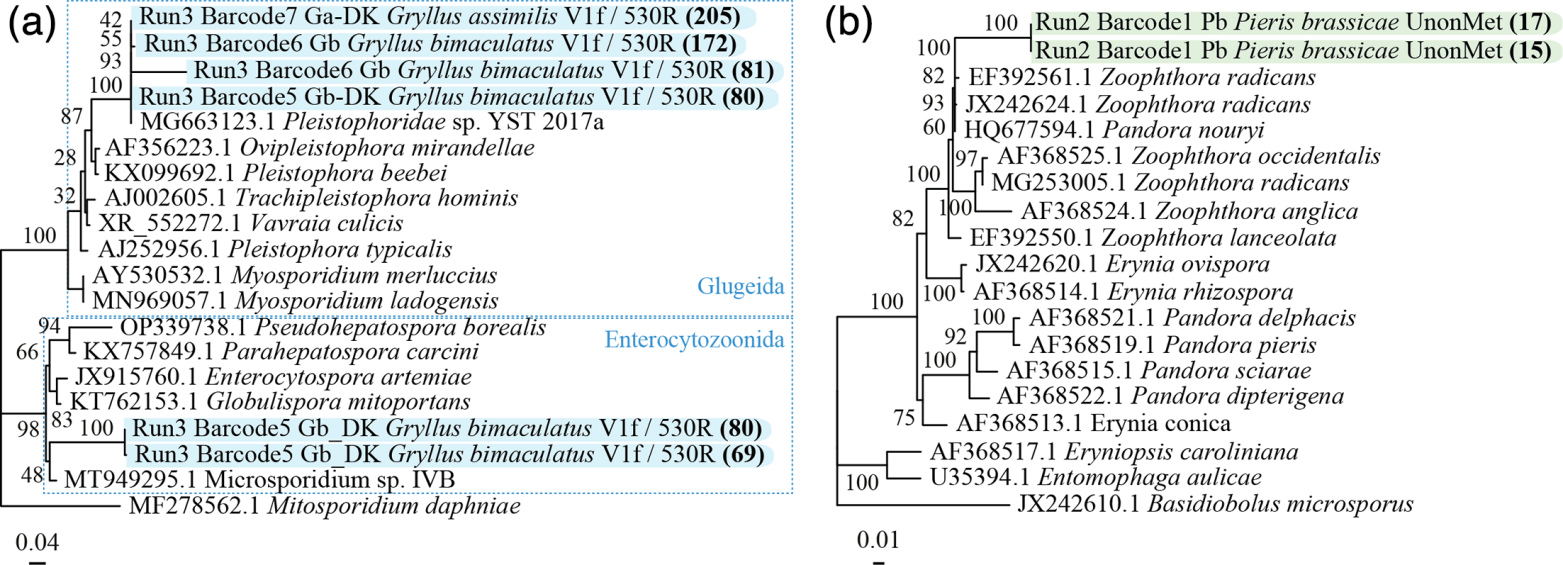
(a) Microsporidia 18S phylogenetic tree. When the same species is represented several times, its sampling location differed. ‘DK’ was sampled in Denmark and the rest in the UK. Tips depicting amplicons from the same run for *G. bimaculatus* barcode 5 indicate a potential co-infection. (**b**) Fungi 18S phylogenetic tree. *Furia* have been renamed to *Pandora* as these were recently described to be synonymous [72]. The trees were constructed using the GTR model, and a bootstrap analysis was performed with 1,000 replicates. The number of reads supporting the different consensus sequences used for phylogenetic analyses is shown in bold and in parentheses.

Four microsporidian consensus sequences were recovered using V1F/530R, supported by 69–205 reads (Amplicons Table). Most clustered with *Albopleistophora grylli* (Pleistophoridae sp. YST), a known microsporidian species infecting *G. bimaculatus* and *G. assimilis* [54,55], with 100% bootstrap support (Fig. 1a). Another sequence clustered with Microsporidium sp. IVB, which was derived from *G. bimaculatus* tissue (98% bootstrap support), previously isolated from *Gammarus* spp., suggesting a potential co-infection or incidental ingestion of spores through contaminated feed.

### Fungi

Multiple yeast and fungal taxa were recovered, including genera such as *Debaryomyces*, *Hyphopichia*, *Tetrapisispora*, *Kodamaea* and *Penicillium*. While most of these are common organisms and not typically associated with insect pathology, two noteworthy fungal amplicon consensus sequences, belonging to the Erynioideae (Entomophthoromycotina), were recovered from *Pieris brassicae* using the 574*f/UNonMet_DB primer pair (Fig. 1b) [56]. The SSU amplicon consensuses from this group matched either *Pandora nouryi* or *Zoophthora radicans* (>99% identity), both of which are well documented as entomopathogenic fungi [see blast results in Amplicons Table]. The phylogenetic placement of *P. nouryi* in the SSU tree shows that the species clusters with *Zoophthora* spp., suggesting that the amplicon sequences likely belong to a single *Zoophthora* species infecting *P. brassicae*.

### Gregarines

**Fig. 2.**
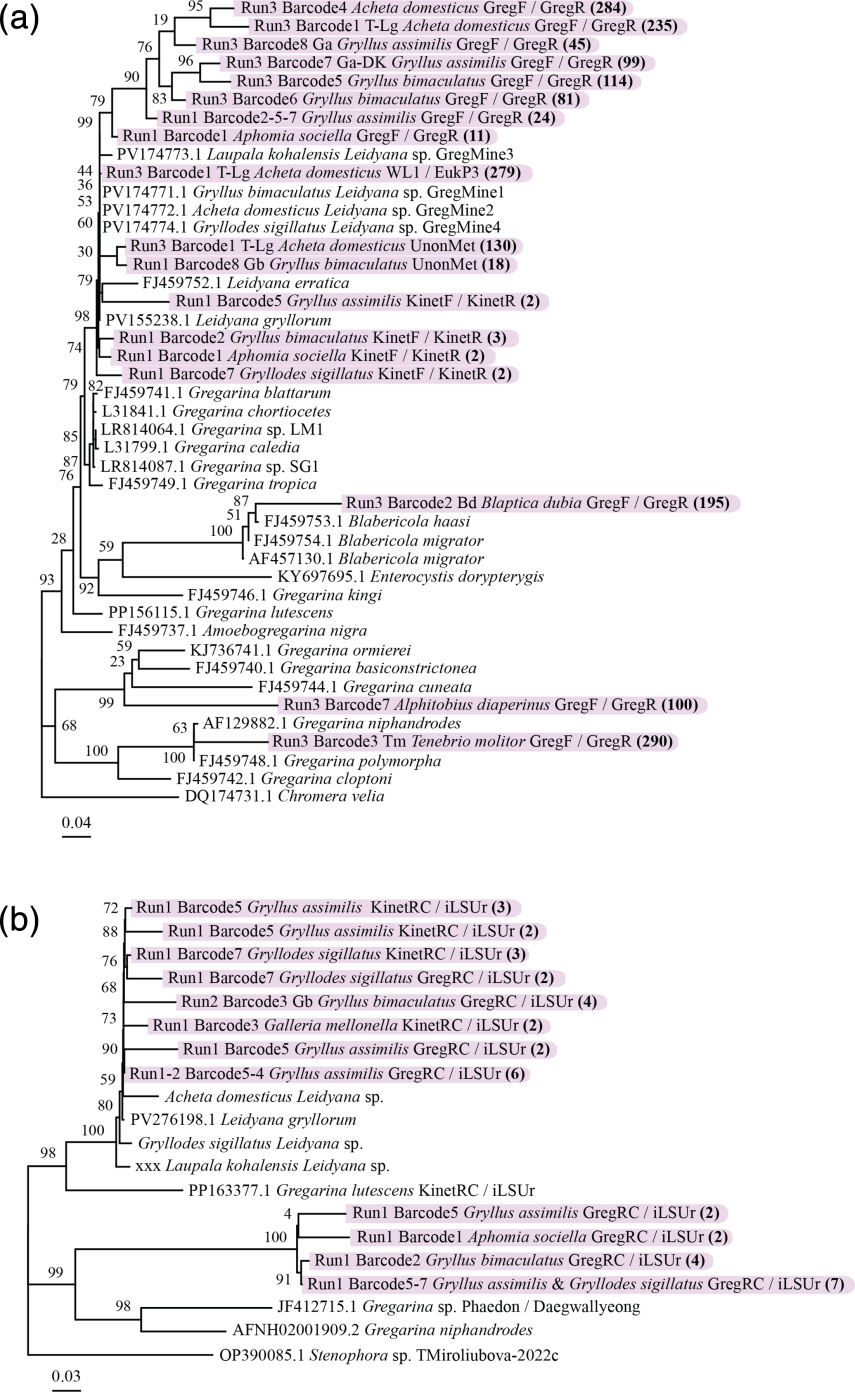
(a) Gregarine 18S and (b) 28S phylogenetic tree. The trees were generated with the GTR model, and a bootstrap analysis was performed with 1,000 replicates. The number of reads supporting the different consensus sequences used for phylogenetic analyses is shown in bold and in parentheses.

With regard to gregarines, SSU phylogenetic analysis confirmed expected placements. A consensus sequence from *Blaptica dubia* clustered with *Blabericola migrator*, a gregarine infecting blaberid cockroaches. Another sequence from *T. molitor* had 98.28% similarity to *Gregarina polymorpha*, its known gregarine parasite (Fig. 2) [57].

Amplicons from *A. diaperinus* clustered within a clade comprising *Gregarina basiconstrictonea* and *Gregarina ormierei*, parasites of darkling beetles (Tenebrionidae) [58]. The tree topology and sequence similarities suggest that *A. diaperinus* consensus sequences are representing a species lacking molecular data to date. It could be *Gregarina alphitobii*, previously described, solely based on morphology [59].

Another interesting clade is the one encompassing all the cricket (Gryllidae) amplicon consensuses (Fig. 2a). This clade includes the references of *Leidyana erratica* and *Leidyana gryllorum*, the latter having been published recently (GenBank accessions FJ459752 and PV155238, respectively). An amplicon consensus from *A. sociella* falling within this clade suggests possible contamination (i.e. from the host during handling in the lab, during sample pooling or barcode demultiplexing). Past work has also shown that gregarine oocysts can be volatile and could easily contaminate a lab environment [60,61].

**Fig. 3.**
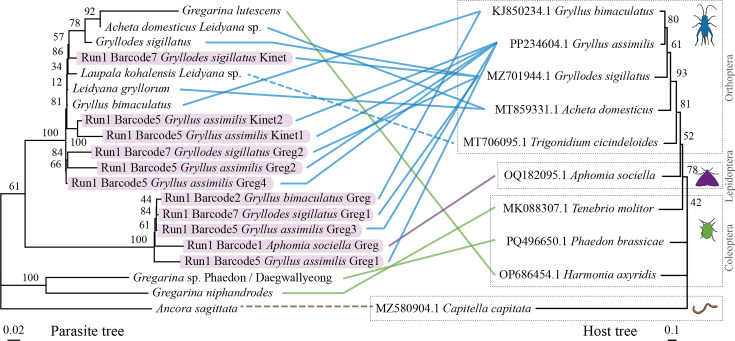
Gregarine–host co-phylogenetic relationship. The left phylogeny depicts the gregarine phylogenetic tree performed with concatenated 18S and 28S sequences. The phylogeny on the right is a rooted maximum likelihood tree of the gregarine–host cytochrome c oxidase subunit I (COI) gene based on nucleotide sequences recovered from GenBank. Hosts are highlighted with boxes and silhouette images (modified from https://www.phylopic.org/). The outgroup *Capitella capitata* was selected as it is the host of the gregarine phylogeny outgroup (*Ancora sagittata*). As there is no available COI gene for *Laupala kohalensis*, the COI gene of the close relative *Trigonidium cicindeloides* was used. The lines between the two phylogenies depict the gregarine–host relationship, with the colours indicating host orders.

Long-read sequencing of gregarine SSU-LSU regions yielded 11 consensus sequences. Two clusters were identified (Fig. 2b). The first is formed with *L. gryllorum,* while the other lacks an available reference. Gregarine and host co-phylogenetic trees support the divergence of a potential new gregarine clade infecting cricket hosts (Fig. 3).

## Discussion

Primer design for metabarcoding eukaryotic endosymbionts is challenging, requiring specificity for target parasites while minimizing host DNA amplification [21,29]. The V4 region of the 18S gene is often selected to reduce off-target amplification [21,62, 63]. *In silico* tools like TestPrime [64] assess primer specificity, but our results emphasize that *in situ* validation through PCR remains essential for reliability and to demonstrate how they perform on samples that contain a complex mix of organisms. For example, Amoebozoan primers only retrieved insect DNA in this study.

The GregF/GregR primer set recovered more gregarine sequences than previously published primers, demonstrating its potential efficiency and high specificity. The non-metazoan primer 574*f/UNonMet_DB successfully amplified non-metazoan DNA but resulted in few protistan sequences and lower diversity of gregarines than the GregF/GregR primer set. One possible optimization for this primer pair is adjusting the annealing temperature, which can influence which organisms are successfully amplified, but this requires specific optimization for each taxon, which is labour intensive, and results can be unpredictable when working on taxa with non-previous molecular data [21]. The AmoebozoaF/AmoebozoaR and V1F/CM-V5R primers had poor specificity, amplifying insect, bacterial and fungal DNA [see Amplicons Table]. Balancing amplification efficiency and specificity remains a key challenge.

Primers yielding shorter reads, like GregF/GregR, could still recover diverse and novel sequences even in the presence of host DNA. This study highlights a scarcity of gregarine data in reference databases and phylogenies [8], especially from crickets. Previous studies reported potential cross-infections among gregarines infecting Gryllidae [65]. However, more recent investigations that integrate both morphological and molecular evidence suggest that gregarines may in fact exhibit a narrower host range, being limited to a single host species. In these newer studies, what was once interpreted as cross-infection may instead represent phenotypic plasticity, where variations in gregarine morphology are driven by host subspecies differences and geographical factors, which likely biassed earlier morphology-based assessments [66,67]. Our co-phylogenetic analysis (Fig. 3) revealed a potential novel Gryllidae-infecting clade that will need further research to fully describe these gregarines. Our study also provides one of the first molecular identifications of a gregarine infecting *A. diaperinus*, closely related to *G. basiconstrictonea* and *G. ormierei*, known from tenebrionid and scarabaeid beetles [67,68]. However, the 84.75% identity with our sequence suggests that a different species, possibly *G. alphitobii* as described by Bala *et al*. [59], might be infecting *A. diaperinus*.

Regarding fungal organisms, a potential *Zoophthora* sp. was detected in *P. brassicae* using the 574*f/UNonMet_DB primer pair, suggesting a natural or accidental infection. The SSU phylogenetic analysis clustered the consensus sequences with *Z. radicans*, a well-known entomopathogen associated with insect epizootics [69]. Such fungi could potentially help control *P. brassicae* populations in organic farms, reducing economic losses to Brassica crops. Interestingly, although *P. nouryi* was initially described and isolated from aphid hosts [70,71], systematic studies on its taxonomic status are scarce. This may explain its unexpected clustering and sequence similarity with *Z. radicans* (99.7% identity between *P. nouryi* accession HQ677594 and *Z. radicans* accession MG252997). The Erynioideae subfamily, which includes the genera *Zoophthora*, *Erynia*, *Furia* (recently synonymized with *Pandora* [72]) and *Pandora*, shows taxonomic inconsistencies; while *Zoophthora* is monophyletic, the other three genera are polyphyletic and in need of revision based on molecular data [73,75]. Regarding the fungi sister clade Microsporidia, the V1F/530R primer detected a potential co-infection in *G. bimaculatus*, reinforcing its effectiveness. The detection of *A. grylli* (Pleistophoridae sp.) in this cricket host species has been recently described [55], but a potential infection by an Enterocytozoonida will require further investigation.

This study highlights the importance of primer validation for accurate microeukaryote metabarcoding in reared insects and other hosts. By combining a cocktail of group‐specific primers with an amplicon pooling strategy and Nanopore barcoding, we achieved a high‐throughput, multiplexed approach that overcomes the limitations of traditional PCR assays targeting microeukaryote pathogens. This integrated method not only reduces time and cost compared to conventional techniques and Illumina-based approaches [29], but it also enhances the resolution of parasite systematics and improves diagnostic accuracy for detecting protist and microsporidian pathogens. Although similar high‐throughput amplicon sequencing methods have been applied to broader eukaryotic diversity surveys [76], our application to reared insect systems is relatively unique. Moreover, the ongoing need for more accurate DNA barcodes to generate well‑supported phylogenies makes the development of gregarine‑specific primers (GregF/GregR) a significant advancement, as this economically important group was previously absent from major primer databases. [10,22]. Our detection of novel gregarine lineages in crickets and the first molecular characterization of gregarines in *A. diaperinus* demonstrates the capacity of this approach to uncover hidden pathogen diversity with direct applications for pathogen surveillance in the growing insect farming industry.

The amplicon pooling strategy presented here can be readily adapted to other host–parasite systems and expanded to include additional primer sets targeting neglected groups such as Amoebozoa or Kinetoplastea. By combining practical diagnostics with phylogenetically informative data, this Nanopore-based approach provides a valuable tool for both improved biosecurity measures in insect mass-rearing systems and advancing our understanding of protist evolution and host–parasite relationships.

## Supplementary material

10.1099/mgen.0.001649 Uncited Supplementary Material 3.

## Abbreviations

GTR: general time reversible
LSU: large subunit
SSU: small subunit
TGS: third-generation sequencing
