# Supplementary material for: High-throughput detection of microeukaryotic parasites in insects using Nanopore sequencing
Source: Microb Genom. 2026 Mar 16;12(3):001649. doi: 10.1099/mgen.0.001649 (PMC12991807; doi:10.1099/mgen.0.001649)
Supplement: Uncited Supplementary Material 3. [file mgen-12-01649-s001.pdf]

Supplementary material

Table S1 Summary of characteristics of the newly designed gregarine primers in comparison to found primers in the literature. Designed primer sets targeting gregarines were evaluated against the SILVA database (v.138.1, RefNR) using TestPrime and compared to other known pairs. TestPrime parameters allowed up to four mismatches and at least three perfect matches at the 3' end. TestPrime performs *in silico* PCR to classify sequences as 'match', 'mismatch', or 'no data'. The percentage of 'match' sequences among 'match' and 'mismatch' sequences is provided.

Primer Set	Targeted Domain	Accessions	Match	Mismatch	Match Frequencies (%)
Designed primers GregF/GregR	Gregarinasina	178	83	18	82.2%
	Insecta	5631	2696	780	77.6%
	Bacteria	381535	299	198383	0.2%
	Fungi	9373	4616	2107	68.7%
WL1/EukP3 (Schrével *et al.*, 2016)	Gregarinasina	178	0	166	0.0%
	Insecta	5631	0	5277	0.0%
	Bacteria	381535	0	330559	0.0%
	Fungi	9373	0	8711	0.0%
1-18S-GRF/1222-18S-GRR (83)	Gregarinasina	178	105	62	62.87%
	Insecta	5631	4644	613	88.34%
	Bacteria	381535	57	375210	0.02%
	Fungi	9373	8350	499	94.36%
574f/UNonMet (Bass and del Campo, 2020; Minardi *et al.*, 2022)	Gregarinasina	178	50	128	28.1%
	Insecta	5631	8	5499	0.1%
	Bacteria	381535	38	381490	0.0%
	Fungi	9373	7956	1401	85.0%

Table S2 Summary of the different Nanopore runs' metrics. Reads count and average barcode scores were obtained from data available in Amplicons Table (https://github.com/Edouard94).

* indicates two samples that were inadvertently pooled and barcoded together.

Nanopore Run	Host pool	Reads count	Average barcode score	DNA quantity library
1	*Alphitobius diaperinus*	897	90.3	16.2 ng
	Aphomia sociella	906	90.5	
	Gryllus assimilis	1368	87.7	
	Gryllus bimaculatus	938	89.3	
	Galleria mellonella	871	89.9	
	Gryllus sigillatus	1018	87.6	
	Schistocerca gregaria	2193	88.5	
	Gryllus assimilis	1100	89.6	
	Gryllus bimaculatus	951	88.2	
	Galleria mellonella	1277	88.6	
2	*Alphitobius diaperinus*	15	82.5	1.5 ng
	Gryllus assimilis	40	84.9	
	Gryllus bimaculatus	10	96.3	
	Pieris brassicae	26	90.4	
	Pieris brassicae	105	92.3	
3	*Acheta domesticus*	514883	93.5	566 ng
	Blaptica dubia	127554	93.2	
	Gryllus assimilis	453806	92.6	
	Gryllus bimaculatus	74208	90.9	
	Gryllus bimaculatus	62732	93.9	
	*Gryllus assimilis**	170422	91.7	
	Alphitobius diaperinus gregarine*	170422	91.7	
	Acheta domesticus gregarine	250834	94.4	
	Tenebrio molitor	329431	94.1	

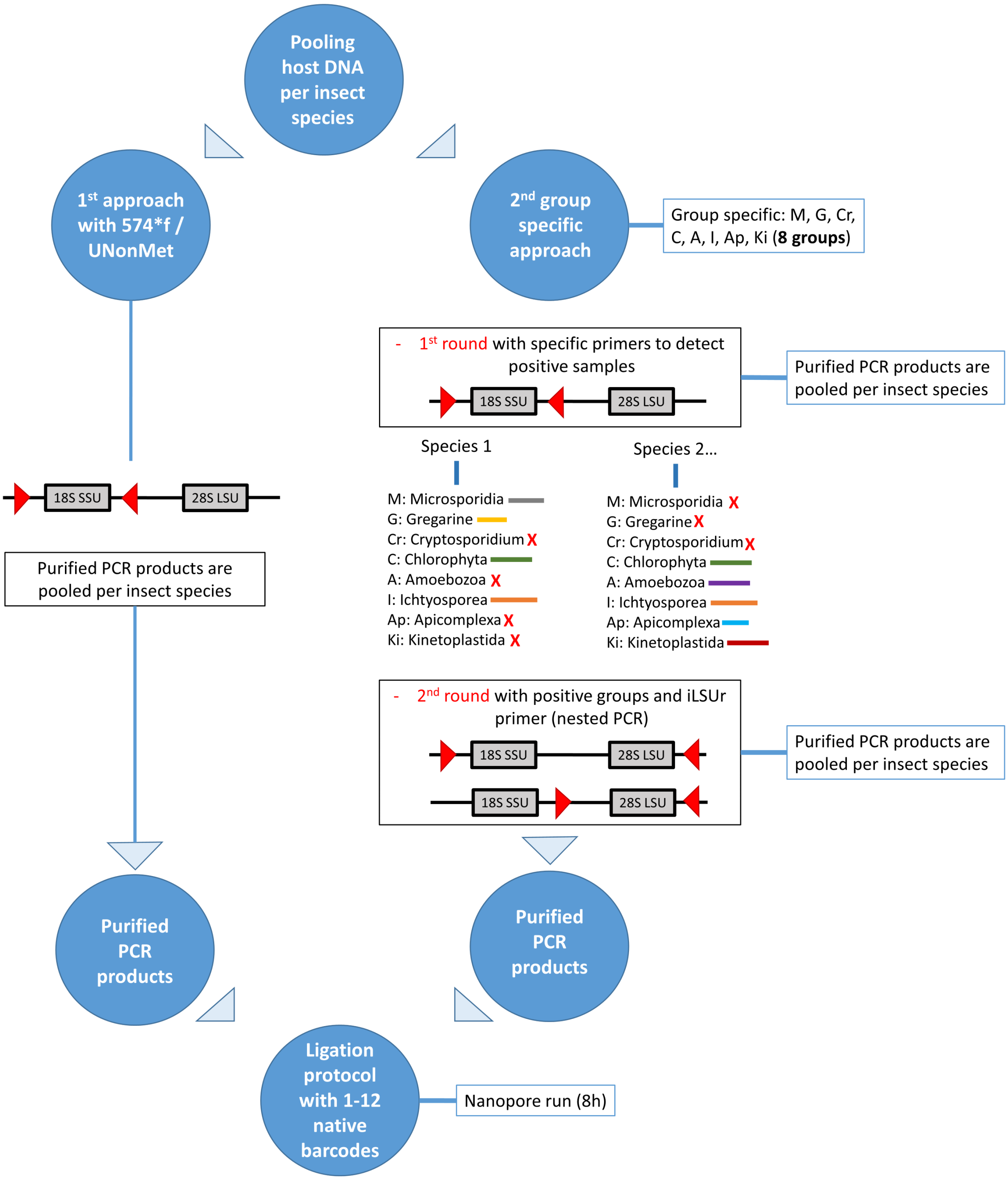

Fig S1 Workflow of the PCR processes used to generate amplicons prior to sequencing. The workflow includes two approaches: the first approach targets the 18S SSU regions using the non-metazoan approach 574*f/UNonMet primers, and the second group-specific approach uses primers for eight groups (Microsporidia, Gregarine, Cryptosporidium, Chlorophyta, Amoebozoa, Ichthyosporea, Apicomplexa, Kinetoplastida) in a two-round PCR process. Purified PCR products are pooled by insect species and ligated with native barcodes before an 8-hour Nanopore sequencing run.

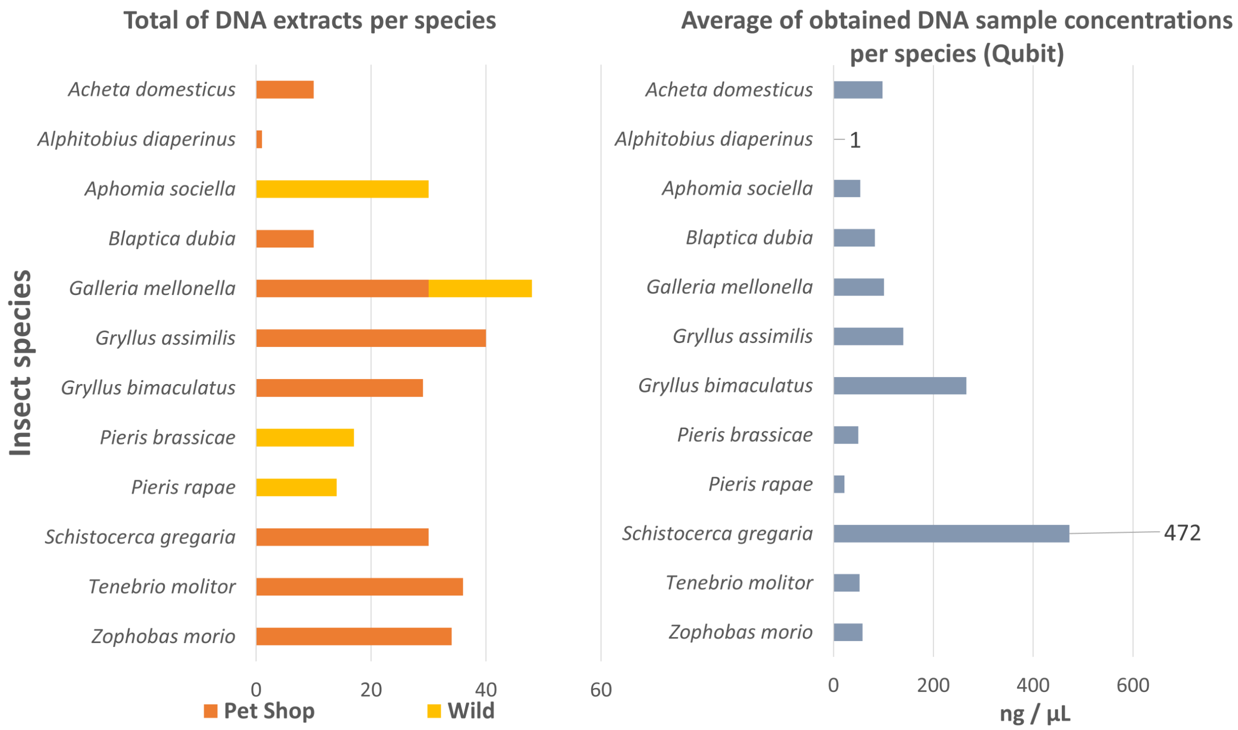

Fig S2 Number of DNA extracts and mean concentration (Qubit) per insect species individual samples. More information about the individual samples can be found here: Samples_Seq_table.xlsx (github.com).

Supplementary file 1: Laboratory notes and PCR gels.

Nanopore experiment lab report for the 1st Run (06/05/2022)

1 Insect pools used during the first run

The insect pools used for the following PCRs were: *P. rapae* (13.5 ng/µL), *A. sociella* (11.3 ng/µL), *G. bimaculatus* (12.6 ng/µL), *Z. morio* (8.28 ng/µL), *T. molitor* (9.27 ng/µL), *S. gregaria* (14.5 ng/µL), *G. mellonella* (only from pet shops, 12.5 ng/µL), *G. assimilis* (8.72 ng/µL). We added samples from the batch samples *Z. morio* (18) (38.4 ng/µL), *Z. morio* (30) (2 ng/µL), *A. diaperinus* (31), *G. sigillatus* (34) (53 ng/µL); also diluted at 10ng/µL.

2 SSU PCR

A gradient PCR was used with the group specific primers (see the following table) and all the prepared DNA pools.

2.1 PCR reaction

The mix used was:

- 5x Phusion HF 4 µL
- 10 mM dNTPs 0.4 µL
- F primer 1 µL
- R primer 1 µL
- DNA 2 µL (20 ng)
- Phusion 0.2 µL
- Nuclease free water 11.4 µL

The primers used for the first SSU PCR were:

Parasite group	Primer name	Reference	Product (Bp)	Sequence (5′-3’)	Tm Phusion
**Microsporidia**	V1F / V5R	Vossbrinck et al., 1987 / Trzebny et al., 2020	780	F: CACCAGGTTGATTCTGCCTGAC<br>R: TAANCAGCACAMTCCACTC	**59**
**Gregarinida**	GregF / GregR	Designed by Edouard (14/04/21)	c.a. 300	F: CCCTTAGATRRYCTGGGCTGC<br>R: CGTGTTACGACTTCTTC	**56**
**Cryptosporidia**	SCL2 / SCR2	Coupe et al., 2005	200	F: CAGTTATAGTTTACTTGATAATC<br>R: CAATACCCTACCGTCTAAAG	**58**
**Chlorophyta**	MGF/ MGR	Mancera et al., 2012	569	F: AGGATAGAGGCCTACCATGGTTTCAA<br>R: CCCCGACTGTCCCTCTCCAT	**69**
**Amoebozoa**	AmoebozoaF / AmoebozoaR	Cannon et al., 2018	360–420	F: GAATTGACGGAAGGGCACAC<br>R: CCAAGAYRTCTAAGGGCATCAC	**61**
**Ichthyosporea**	500F / 900R	Lord et al., 2012	470 - 509	F: CGGCTACCACTTCTACGGAGG<br>R: ATTAACGCCCCCAACTATCCC	**66**
**Apicomplexa**	Apicomplexa B	Cannon et al., 2018	228 – 244	F: TGYGTTTGAATACTAYAGCATGG<br>R: TCTGATCGTCTTCACTCCCTT	**61**
**Kinetoplastea**	Kineto14F / kineto2026R	von der Heyden and Cavalier-Smith (2005)	1·9 – 2·2 kb	F: CTGCCAGTAGTCATATGCTTGTTTCAAGGA<br>R: GATCCTTCTGCAGGTTCACCTACAGCT<br>R: RACTACGAGCTTTTTAACTGC	**70**
**Universal primers**	574*f / UNonMet DB	Minardi (2021)	c.a. 574 (median length)	F: CGGTAAYTCCAGCTCYV<br>R: CTTTAARTTTCASYCTTGCG	**57**

2.2 PCR program

- Initial Denaturation: 98°C, 30sec

30 cycles:

  - Denaturation 98°C, 10s
  - Anneal. X°C, 30s
  - Extension 72°C, 2min

- Final extension 72°C, 10min

Gradient PCR: target 61°C, gradient 15°C

2.3 PCR template

First round PCR

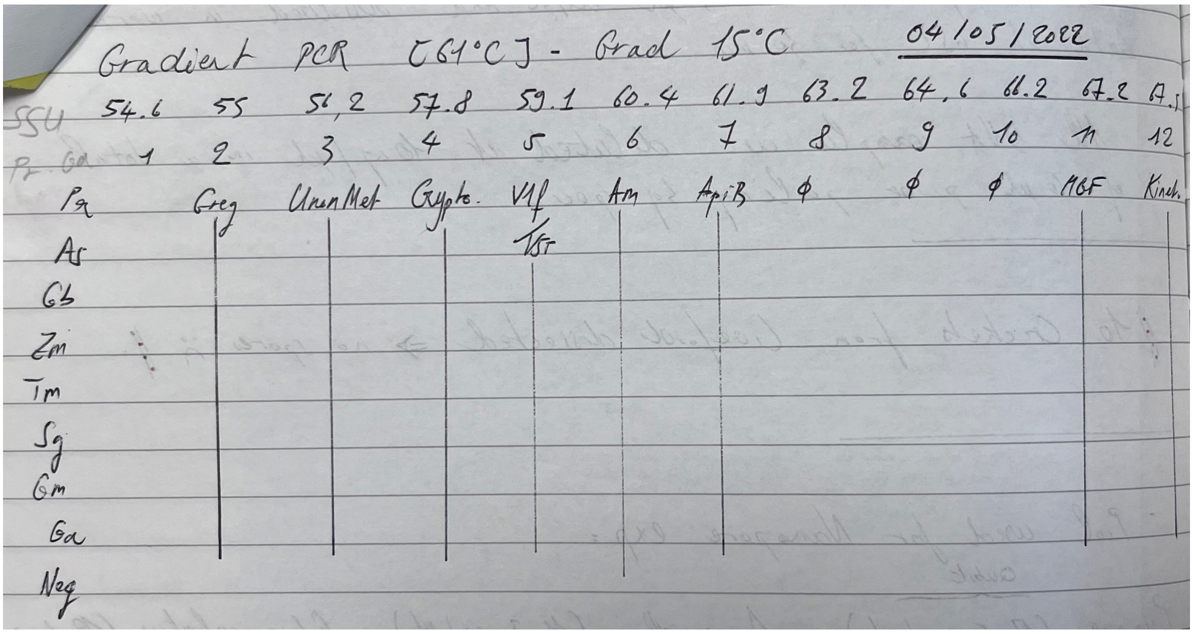

Second SSU PCR to include the other samples and the *Ich* primers

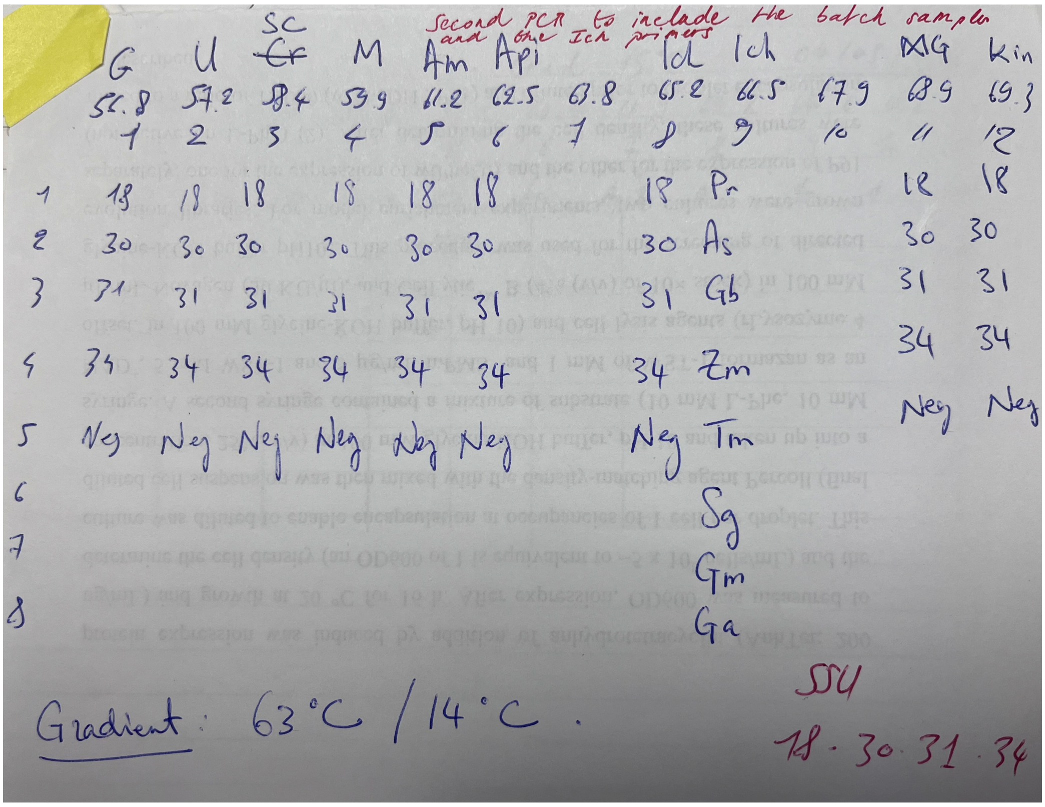

2.4 PCR gels

2.4.1 First SSU PCR gel.

Yellow crosses show positive samples with expected band size. When multiple bands were present, the expected size was excised and purified using the Qiagen Gel PCR Clean-up kit.

PCR gel (1.3%) made the 04/05/2022 Nanopore exp short read (SSU)

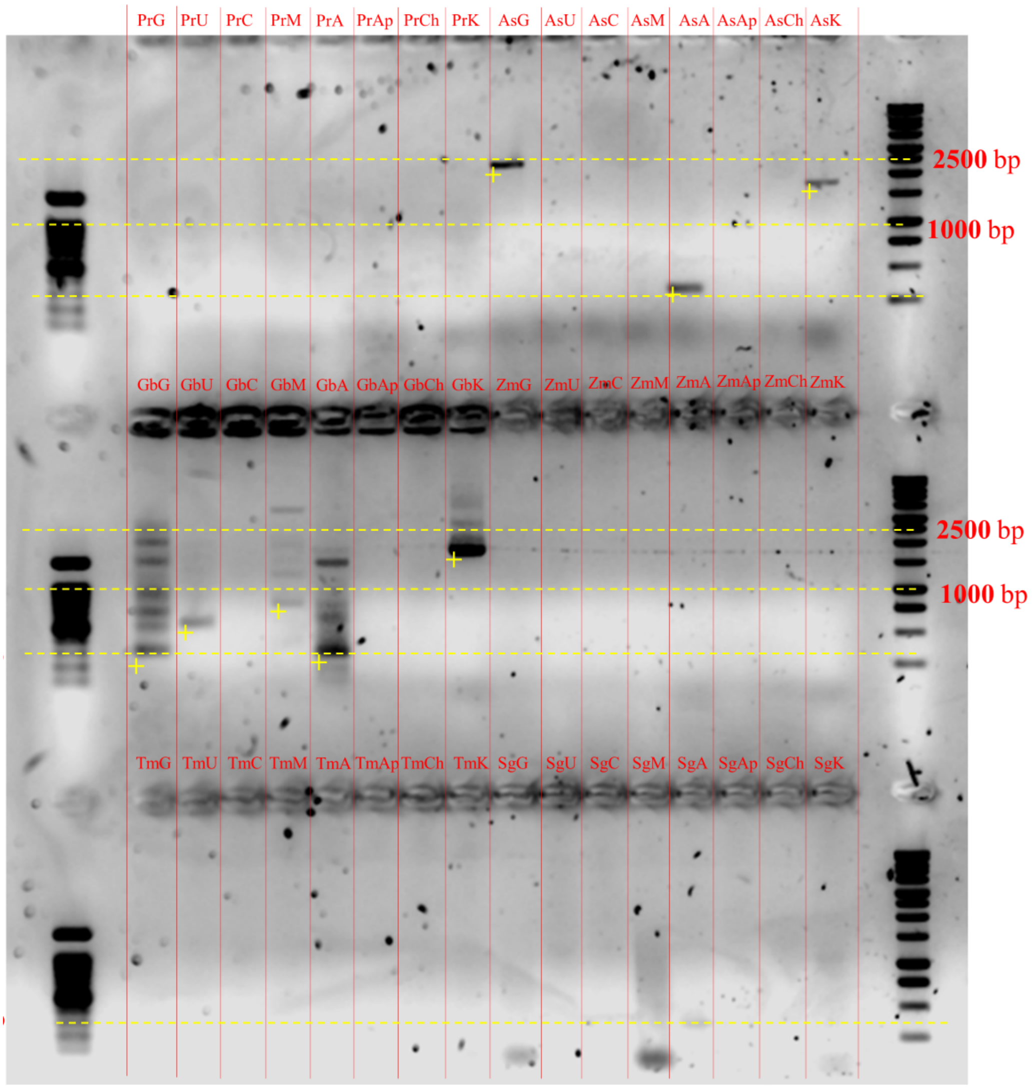

2.4.2 *Second SSU gel*

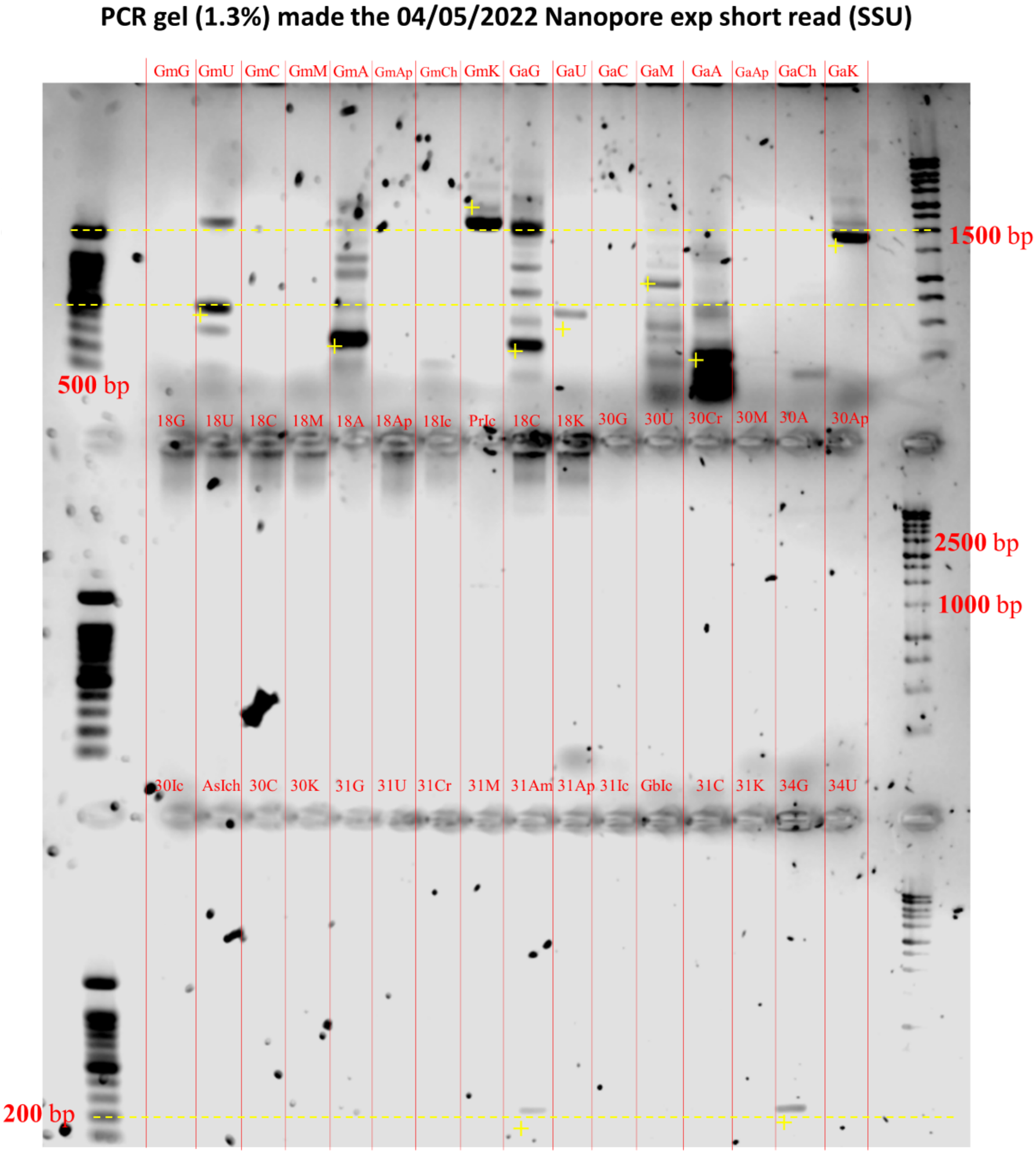

2.4.3 *Third SSU gel*

PCR gel (1.3%) made the 04/05/2022 Nanopore exp short read (SSU)

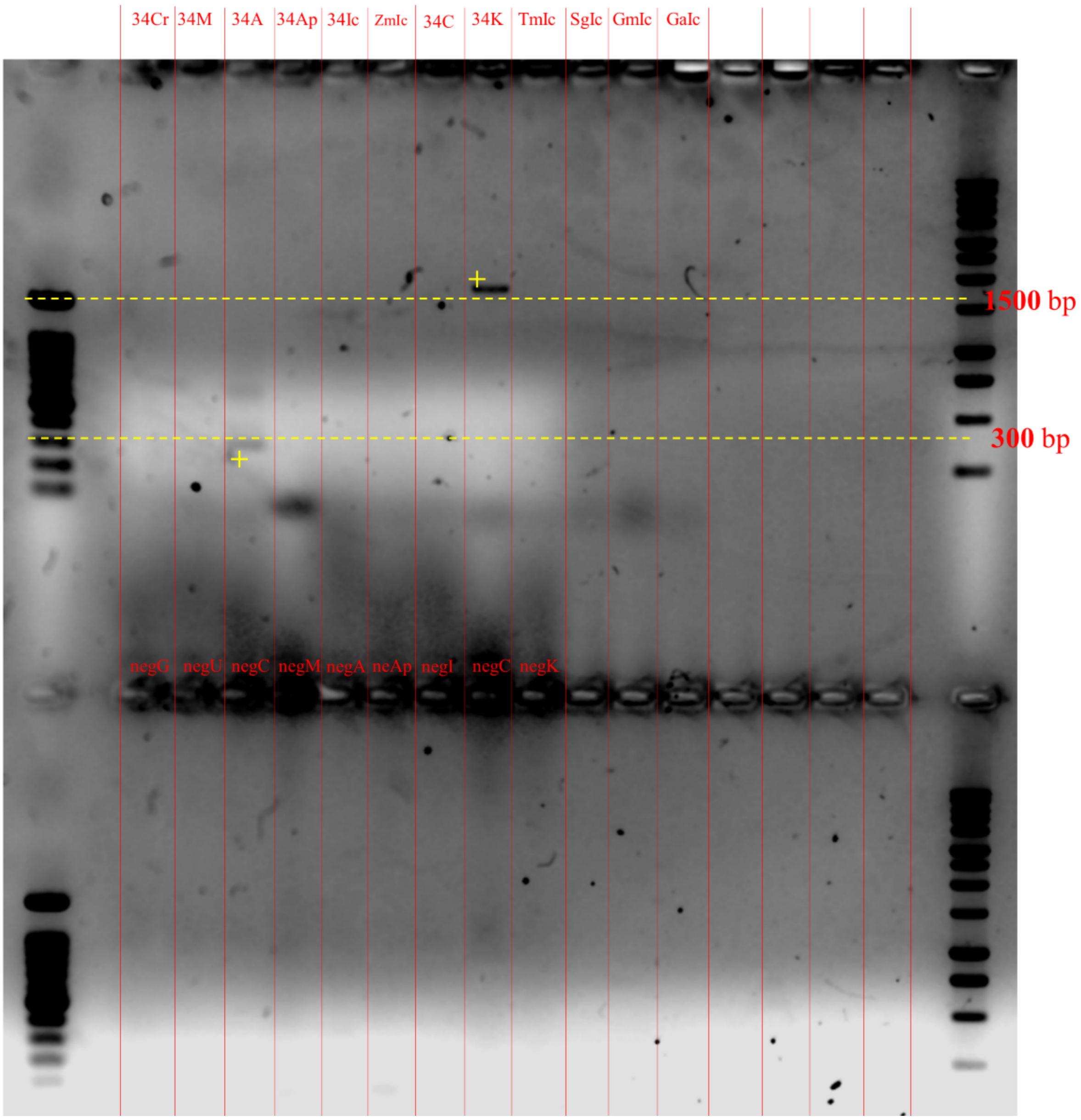

3 Long LSU PCR

PCRs targeting the 28S were performed with insect DNA pools that showed expected PCR products from the previous SSU specific PCRs. The following table shows which pools were positive for each primer group:

		Pr	As	Gb	Zm	Tm	Sg	Gm	Ga	18 (Zm)	30 (Zm)	31 (A. diapernus)	34 (G. sigillatus)
	M			+					+				
	Greg		+	+					+				+
	Api												
	Cr												
	Kinet		+	+				+	+				+
	Chloro												
	Am		+	+			+	+	+			+	+
	Ichtyo												

For the first long PCR, a universal broad reverse primer was used (iLSUr) as well as the specific forward primer used during the first SSU PCRs. The new set of primers was:

Parasite group	Primer name	Reference	Product (Bp)	Sequence (5′-3’)	Tm Phusion
**Microsporidia**	V5F / iLSUr	Trzebny et al., 2020 / Xu 2010	2800 - 3000	F: GATTAGANACCNNNGTAGTTC	**54**
				R: ACCTGTCTCACGACGGTCTAAAC	
**Gregarinida**	GregF / iLSUr	Xu 2010	3300 - 4000	F: CCCTTAGATRRYCTGGGCTGC	**64**
				R: ACCTGTCTCACGACGGTCTAAAC	
**Amoebozoa**	AmoebozoaF / iLSUr	Xu 2010	c.a. 4500	F: GAATTGACGGAAGGGCACAC	**65**
				R: ACCTGTCTCACGACGGTCTAAAC	
**Kinetoplastea**	Kineto14F / iLSUr	Xu 2010	6000 - 7000	F: CTGCCAGTAGTCATATGCTTGTTTCAAGGA	**67**
				R: ACCTGTCTCACGACGGTCTAAAC	

3.1 PCR reaction

- 5x Phusion HF 4 µL
- 10 mM dNTPs 0.4 µL
- F primer 1 µL
- R primer (iLSUr) 1 µL
- **DNA from the positive pools** 2 µL
- Phusion 0.2 µL
- Nuclease free water 11.4 µL

3.2 PCR program

- Initial Denaturation: 98°C, 30sec

30 cycles:

  - o Denaturation 98°C, 10s
  - o Anneal. X°C, 1min40s
  - o Extension 72°C, 2min30s

- Final extension 72°C, 10min

Gradient PCR: target 61°C, gradient 14°C

3.3 PCR template

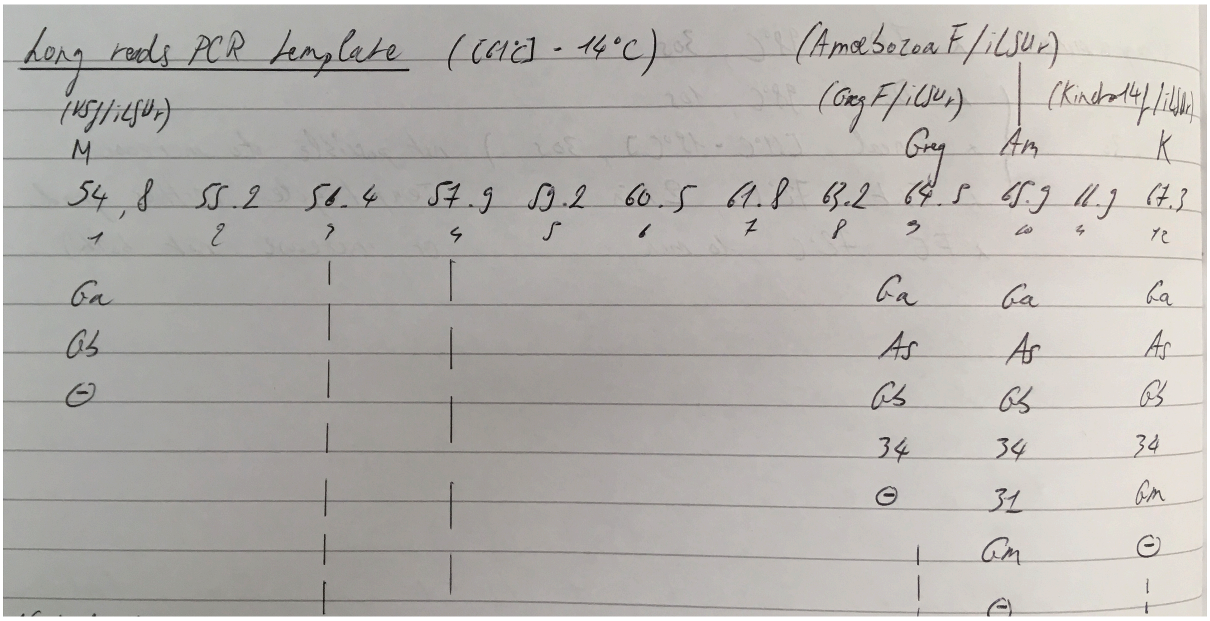

3.4 PCR gel

Yellow crosses show positive samples with expected band size. Blue crosses show expected bands, which are faint. Again when multiple bands were seen the expected one was excised.

PCR gel (1.3%) made the 05/05/2022 Nanopore exp long read (SSU positives)

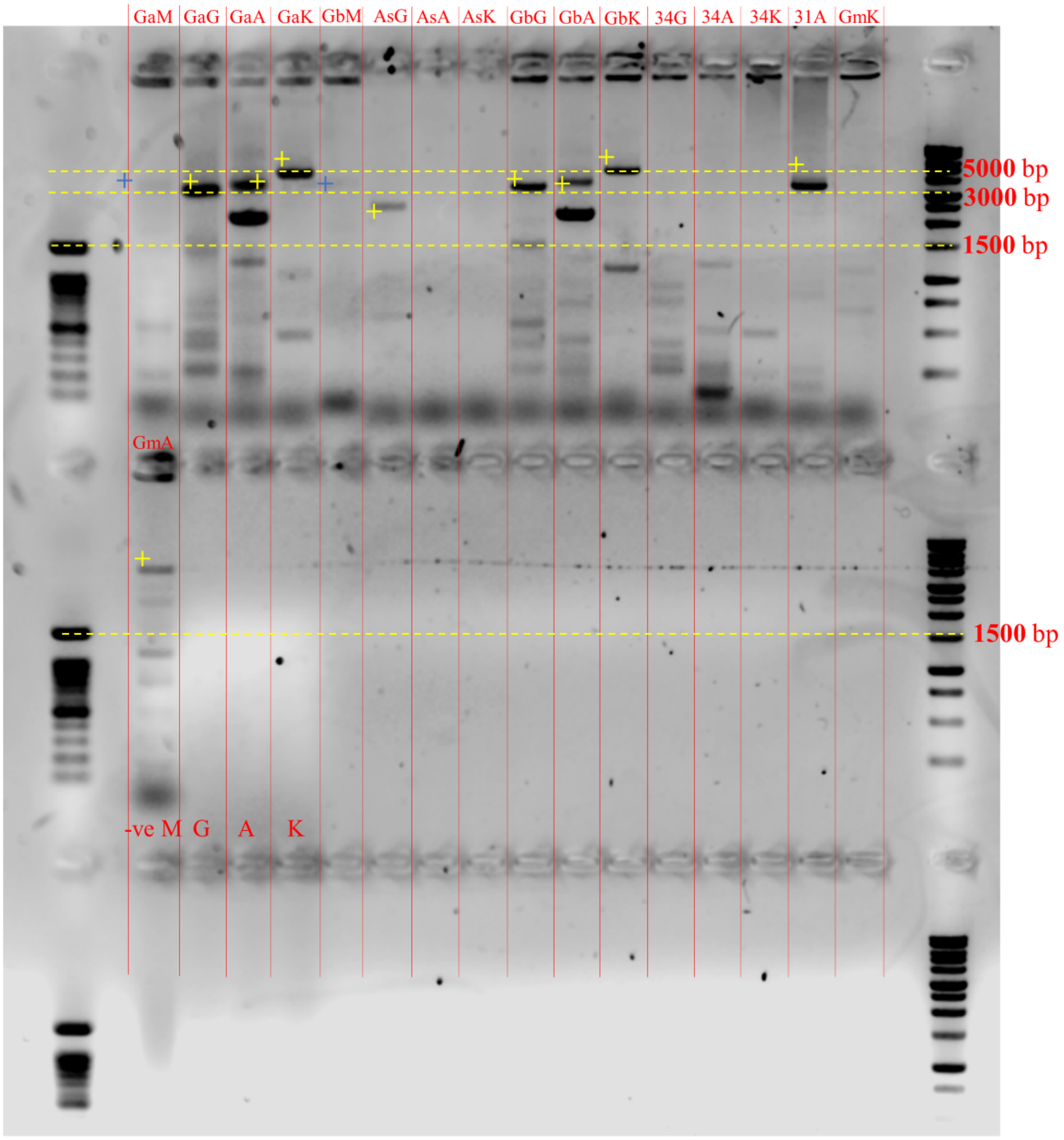

4 Nested PCR

For the nested PCR, the same universal broad reverse primer (iLSUr) was used, and the reverse complementary sequence of the specific reverse primer (now becoming a forward primer) was used during the first PCRs. The new set of primers was:

Parasite group	Primer name	Reference	Product (Bp)	Sequence (5′-3’)	Tm Phusion
**Microsporidia**	V5r(rc) / iLSUr	Trzebny et al., 2020 / Xu 2010	2700 - 2900	F: GAGTGGAKTGTGCTGNTTA	**59**
				R: ACCTGTCTCACGACGGTCTAAAC	
**Gregarinida**	GregR(rc) / iLSUr	Xu 2010	3000 - 3700	F rc: GAAGAAGTCGTAACACG	**56**
				R: ACCTGTCTCACGACGGTCTAAAC	
**Amoebozoa**	AmoebozoaR(rc) / iLSUr	Xu 2010	c.a. 4200	F: GTGATGCCCTTAGAYRTCTTGG	**65**
				R: ACCTGTCTCACGACGGTCTAAAC	
**Kinetoplastea**	kineto2026R(rc) / iLSUr	Xu 2010	c.a. 5000	F: AGCTGTAGGTGAACCTGCAGAAGGATC	**68**
				R: ACCTGTCTCACGACGGTCTAAAC	

4.1 PCR Reaction

- 5x Phusion HF 4 µL
- 10 mM dNTPs 0.4 µL
- F primer 1 µL
- R primer (iLSUr) 1 µL
- **Unpurified PCR product from the long reads PCR** 2 µL
    - Every PCR product from the first LSU PCR was used, even those which did not show an expected band
- Phusion 0.2 µL
- Nuclease free water 11.4 µL

4.2 PCR program

- Initial Denaturation: 98°C, 30sec

30 cycles:

- Denaturation 98°C, 10s
- Anneal. X°C, 1min40s
- Extension 72°C, 2min30s

- Final extension 72°C, 10min

Gradient PCR: target 61°C, gradient 14°C

4.3 PCR template

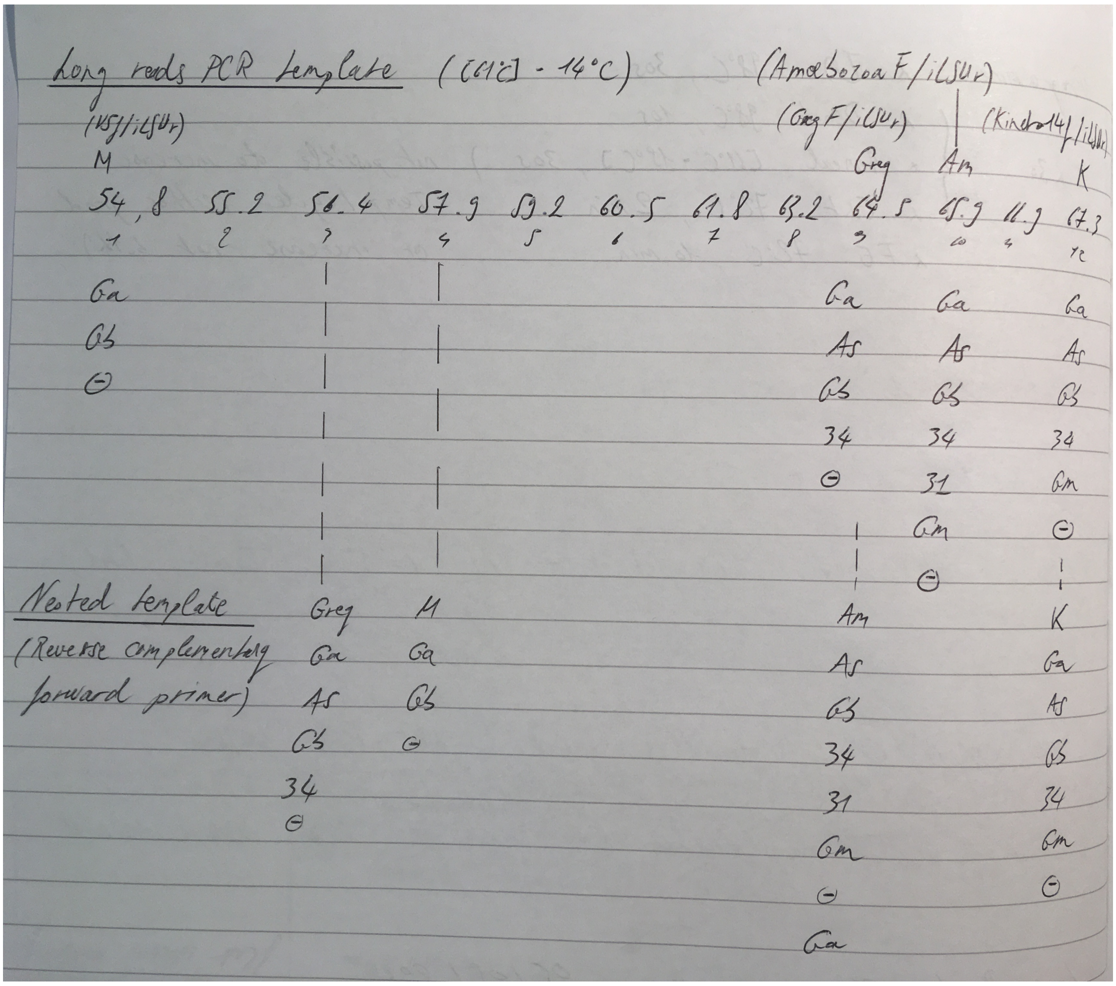

4.4 PCR gel

The gel displayed numerous unexpected bands compared to the initial LSU PCR, partly attributed to the utilisation of unpurified PCR products as templates. Therefore lanes displaying expected DNA band sizes (with expected and faint bands indicated by yellow and blue crosses respectively), above 1.5 kb were excised. The gel within the green boxes denotes what was excised and purified using the Qiagen Gel PCR Clean-up kit.

PCR gel (1.3%) made the 05/05/2022 Nanopore exp long read (nested 'rc' SSU positives)

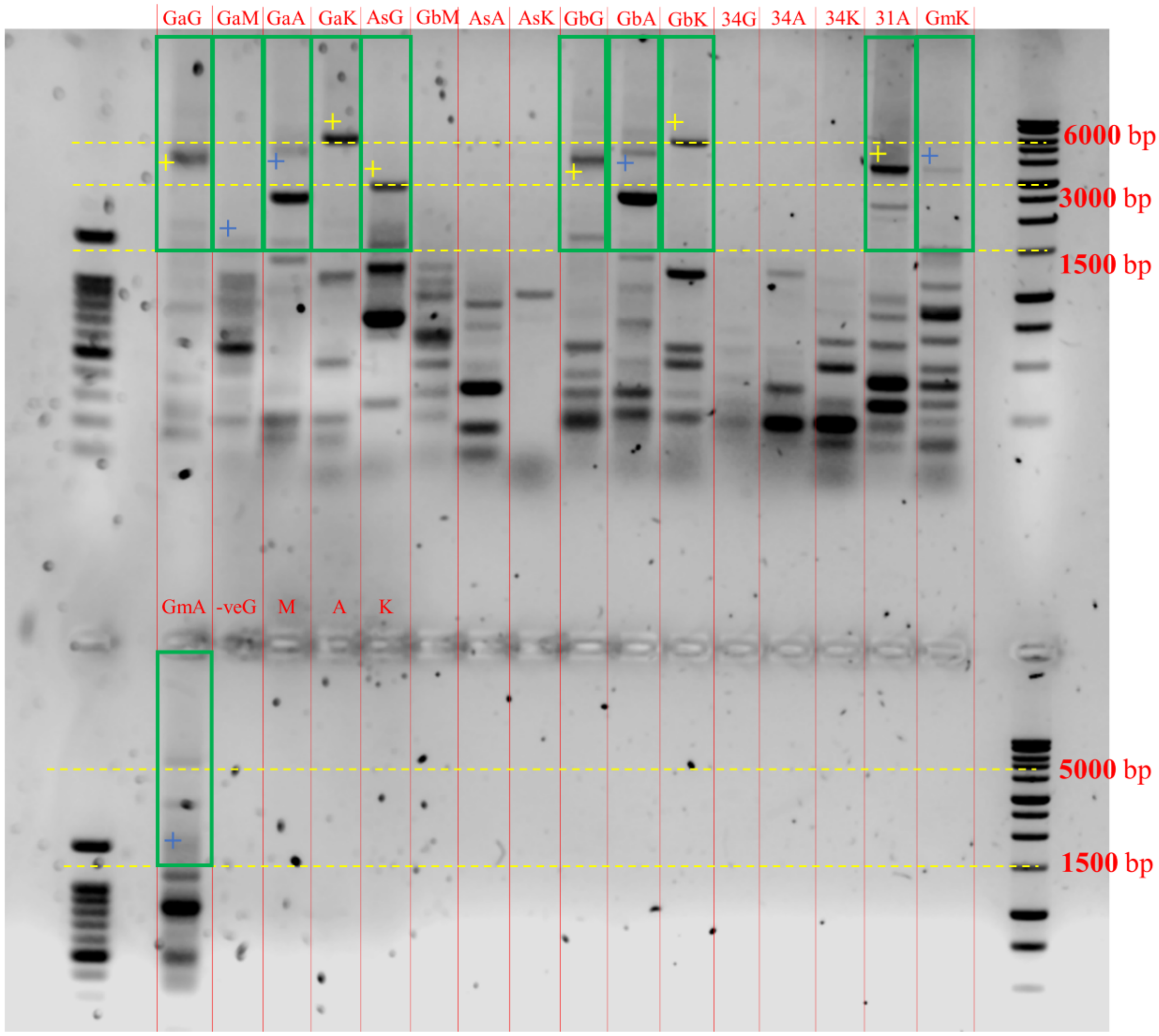

5 Nanopore Run

The PCR band purified products used to perform Nanopore sequencing were the following:

		Pr	As	Gb	Zm	Tm	Sg	Gm	Ga	18 (Zm)	30 (Zm)	31 (A. diapernus)	34 (G. sigillatus)
	UnonMet			+				+	+				
	M			+					+				
	Greg		+	+					+				+
	Api												
	Cr												
	Kinet		+	+				+	+				+
	Chloro												
	Am		+	+			+ (faint band, not selected)	+	+			+	+
	Ichtyo												
	M			+					+				
	Greg		+	+					+				**miss**
	Api												
	Cr												
	Kinet		**miss**	+				**miss**	+				**miss**
	Chloro												
	Am		**miss**	+				+	+			+	**miss**
	Ichtyo												
	M			**miss, under 1.5kb**									
	Greg		+	+					+				**miss, under 1.5kb**
	Api												
	Cr												
	Kinet		**miss**	+				+	+				**miss, under 1.5kb**
	Chloro												
	Am		**miss, under 1.5kb**	+				+	+			+	**miss, under 1.5kb**
	Ichtyo												

The UnonMet samples were kept as individual samples and all the ‘SSU’, ‘long reads’ and ‘long reads nested’ PCR products were pooled by insect species, where 5 µL of each sample/amplicon were used to form the pool. Even the ‘missed’ PCR products that did not show an expected band during the first long reads PCR were added to the pool. For the nested PCR products, only amplicons above 1.5 kbp were excised and purified for sequencing as explained previously.

The Nanopore library was constructed with the **native barcoding amplicons kit EXP-NBD104** and the **ligation kit SQK-LSK109**. Eventually, the following pools of amplicons and respective barcodes were used for sequencing:

Nanopore Run	Sample/Pools	Initial C of pools (ng/uL)	C after End-prep step (ng/uL)	Barcode	Forward sequence	Reverse sequence	C of pooled barcode sample (ng/uL)
05/05/2022	As	9.86	3.94	NB01	CACAAAGACACCGACAACTTTCTT	AAGAAAGTTGTCGGTGTCTTTGTG	1.08
05/05/2022	Gb	28.2	18.3	NB02	ACAGACGACTACAAACGGAATCGA	TCGATTCCGTTTGTAGTCGTCTGT	
05/05/2022	Gm	20.8	12.3	NB03	CCTGGTAACTGGGACACAAGACTC	GAGTCTTGTGTCCCAGTTACCAGG	
05/05/2022	Sg (only SSU)	8.52	1.76	NB04	TAGGGAAACACGATAGAATCCGAA	TTCGGATTCTATCGTGTTTCCCTA	
05/05/2022	Ga	29.4	21	NB05	AAGGTTACACAAACCCTGGACAAG	CTTGTCCAGGGTTTGTGTAACCTT	
05/05/2022	31 (Ad)	10.6	2.52	NB06	GACTACTTTCTGCCTTTGCGAGAA	TTCTCGCAAAGGCAGAAAGTAGTC	
05/05/2022	34 (Gs)	15	6.9	NB07	AAGGATTCATTCCCACGGTAACAC	GTGTTACCGTGGGAATGAATCCTT	
05/05/2022	UnonMet Gb	22.8	5.06	NB08	ACGTAACTTGGTTTGTTCCCTGAA	TTCAGGGAACAAACCAAGTTACGT	
05/05/2022	UnonMet Gm	34.2	7.26	NB09	AACCAAGACTCGCTGTGCCTAGTT	AACTAGGCACAGCGAGTCTTGGTT	
05/05/2022	UnonMet Ga	12.7	1.8	NB10	GAGAGGACAAAGGTTTCAACGCTT	AAGCGTTGAAACCTTTGTCCTCTC	

6 Possible improvements determined after the 1st Nanopore Run

- Do PCR replicates to increase the DNA quantity
- Purified the PCR products between the long reads PCR and the nested one
- Modify the PCR program and increase the number of cycles

Nanopore experiment lab report for the 2nd Run (01/06/2022)

1 SSU PCR

This run focused on sequencing long reads from previous positive samples. New pools were added to the initial ones: *G. mellonella* (wild insects) 12.3 ng/µL; P. brassicae (wild insects) 17.9 ng/µL. First PCR targeting the 18S (SSU) gene of microsporidia and protist parasites were performed on the new pools before further long read PCR.

1.1 PCR reaction

- 5x Phusion HF 4 µL
- 10 mM dNTPs 0.4 µL
- F primer 1 µL
- R primer 1 µL
- DNA 2 µL (i.e., 20ng)
- Phusion 0.2 µL
- Nuclease free water 11.4 µL

1.2 PCR program

- Initial Denaturation: 98°C, 30sec

30 cycles:

  - Denaturation 98°C, 10s
  - Anneal. X°C, 30s
  - Extension 72°C, 2min

- Final extension 72°C, 10min

1.3 PCR template

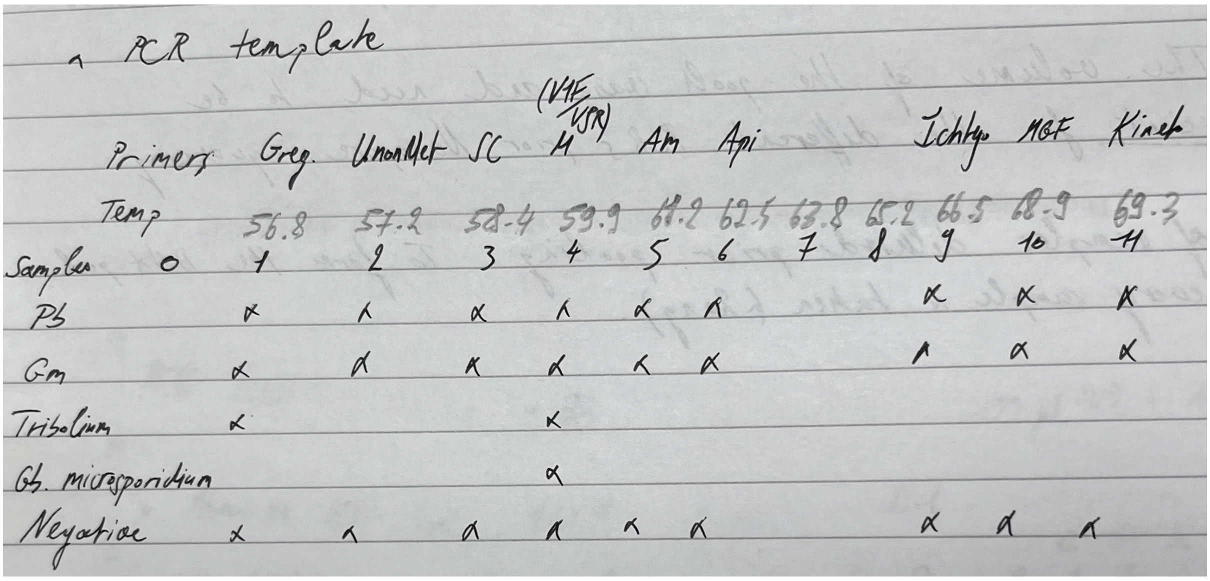

PCR template

Primers		Greg.	UnonMet	SC	M (V1F/V5R)	Am	Api			Ichty.	MGF	Kinet.
Temp		56.8	57.2	58.4	59.9	61.2	62.5	63.8	65.2	66.5	68.9	69.3
Samples	0	1	2	3	4	5	6	7	8	9	10	11
Pb		x	x	x	x	x	x			x	x	x
Gm		x	x	x	x	x	x			x	x	x
Tribolium		x			x							
Gb. microsporidium					x							
Negative		x	x	x	x	x	x			x	x	x

1.4 PCR Gel

Crosses represent excised and purified bands.

PCR gel (1.3%) made the 01/06/2022 SSU amplicons

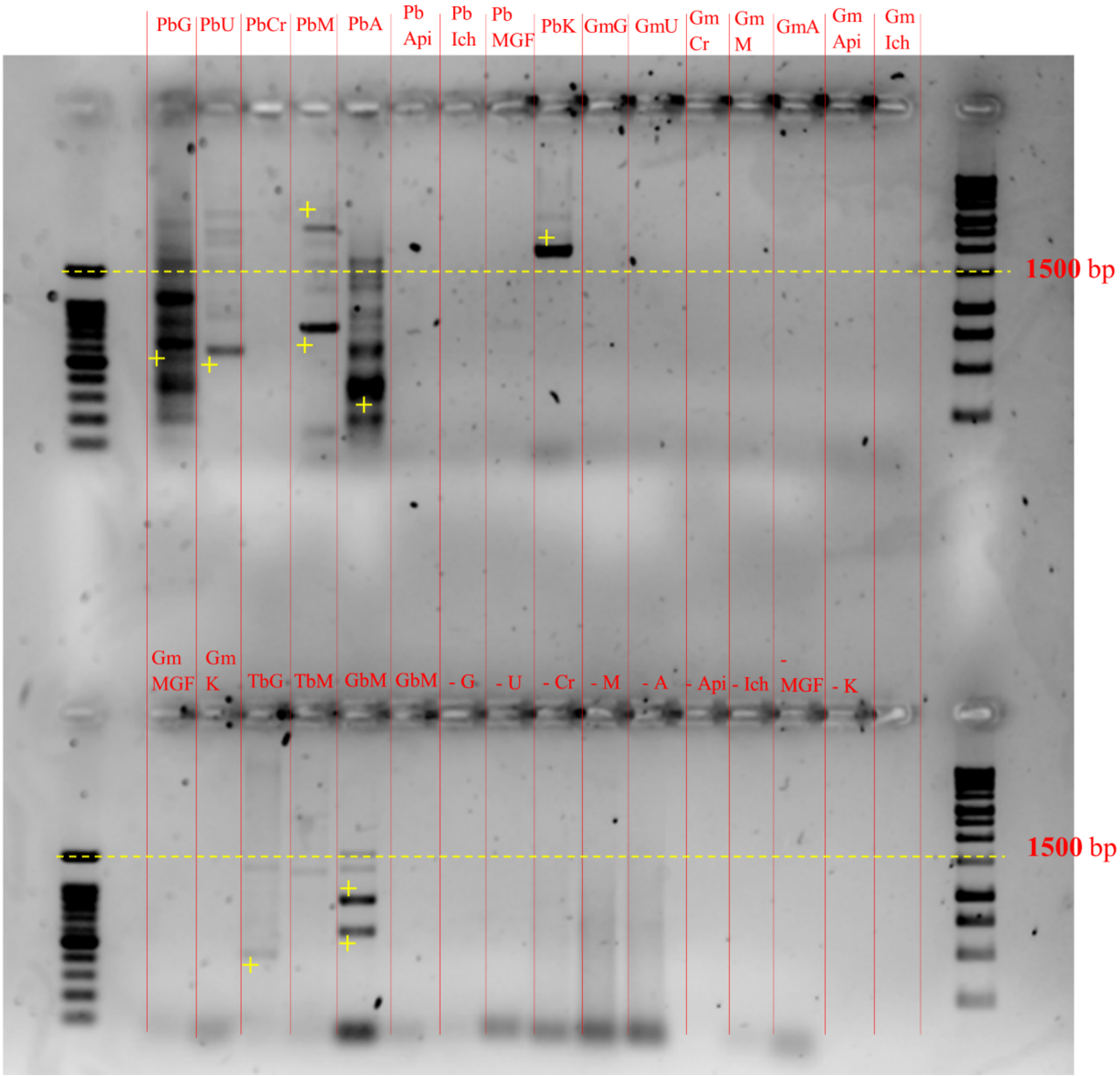

2 Long LSU PCR

The second PCR targeting the 28S gene was made with the DNA pools that showed expected product during the SSU PCR. Interesting positive samples from the 1st run (Ga, Gb, As. 31) were also added to provide more long amplicons reads. PCR reactions (20 µL) were ran in duplicates to have more DNA material.

2.1 PCR reaction

- 5x Phusion HF 4 µL
- 10 mM dNTPs 0.4 µL
- F primer 1 µL
- R primer (iLSUr) 1 µL
- **DNA from the positive pools** 2 µL
- Phusion 0.2 µL
- Nuclease free water 11.4 µL

2.2 PCR program

- Initial Denaturation: 98°C, 30sec

30 cycles:

  - Denaturation 98°C, 10s
  - Anneal. X°C, 1min40s
  - Extension 72°C, 2min30s

- Final extension 72°C, 10min

Gradient PCR: target 61°C, gradient 14°C

2.3 PCR template

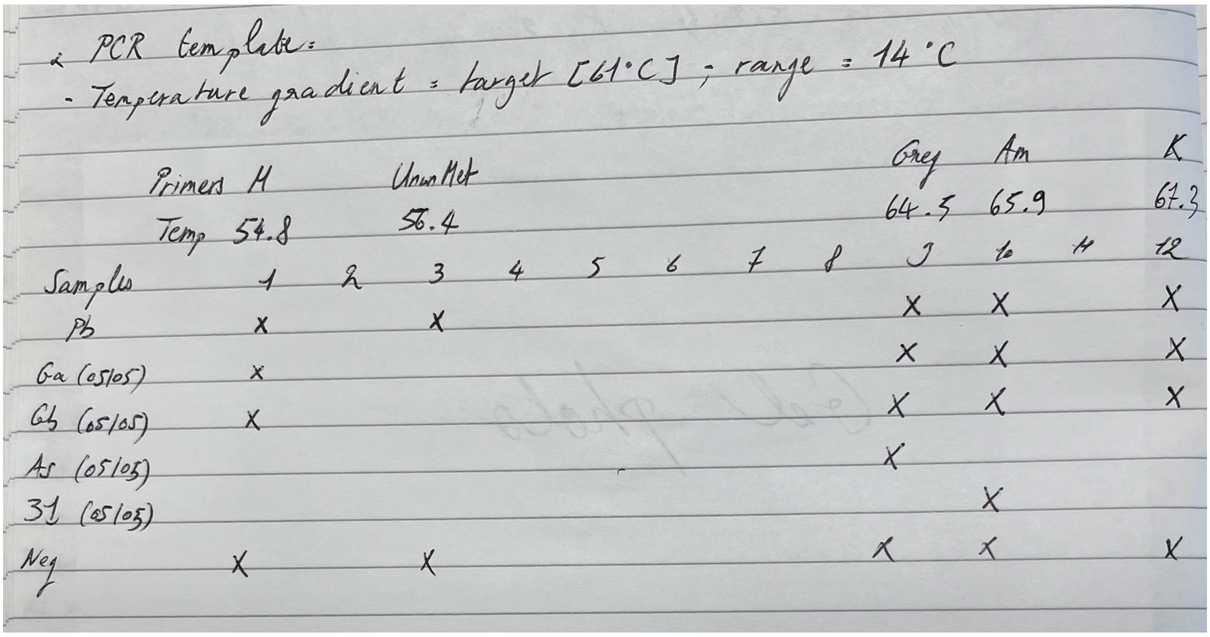

2. PCR template:

- Temperature gradient = target [61°C] ; range = 14 °C

Primers	M		UnmMet						Grey	Am		K
Temp	54.8		56.4						64.5	65.9		67.3
Samples	1	2	3	4	5	6	7	8	9	10	11	12
Pb	X		X						X	X		X
Ga (05/05)	X								X	X		X
Gb (05/05)	X								X	X		X
As (05/05)									X			
31 (05/05)										X		
Neg	X		X						X	X		X

2.4 PCR gel

Crosses represent bands that were excised. Eventually positive PCR reactions were purified and duplicates were pooled together (prior purification).

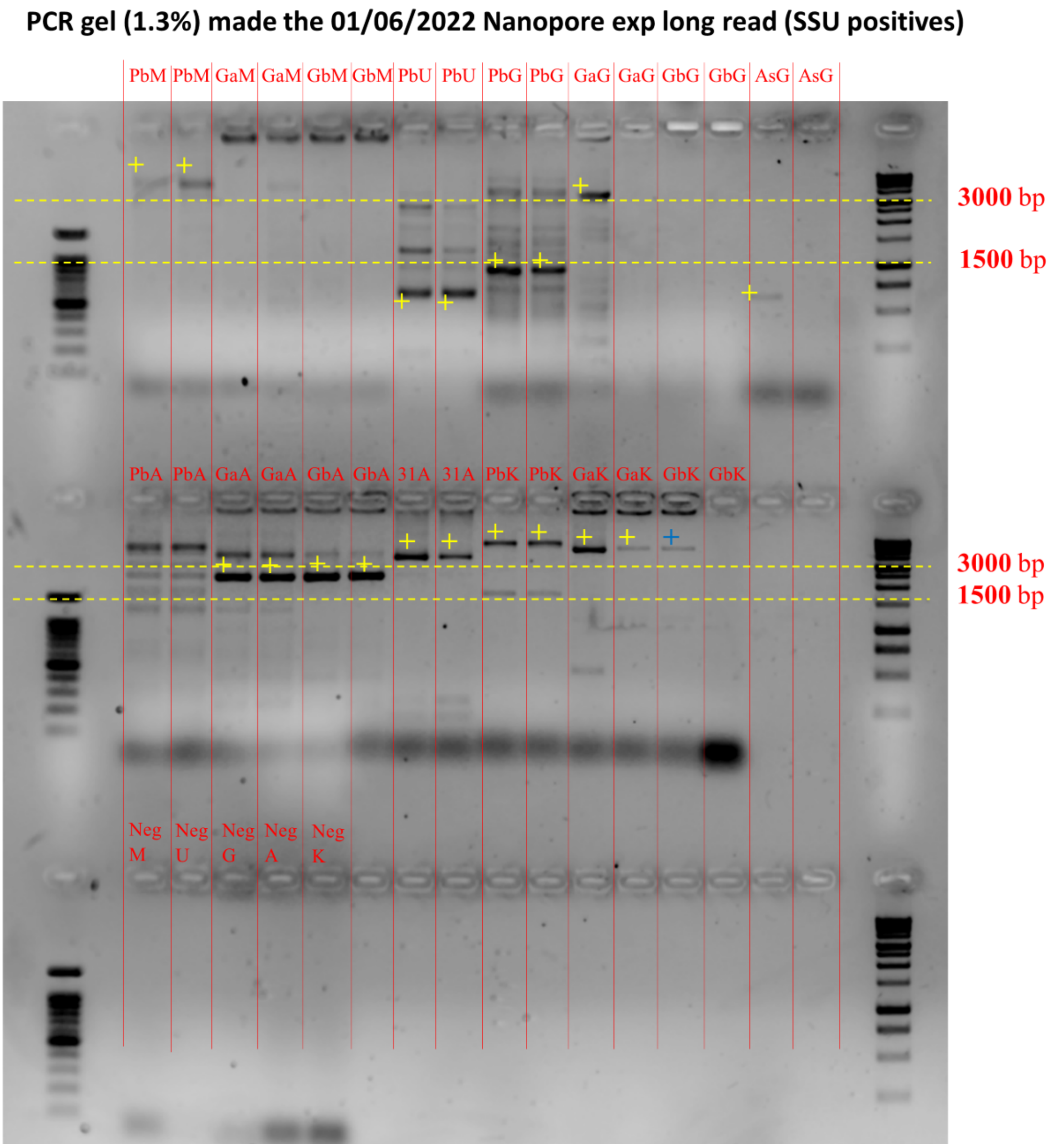

3 Nested PCR

3.1 PCR reaction

- 5x Phusion HF 4 µL
- 10 mM dNTPs 0.4 µL
- F primer 1 µL
- R primer (iLSUr) 1 µL
- **Purified PCR product from LSU PCR** 2 µL
- Phusion 0.2 µL
- Nuclease free water 11.4 µL

3.2 PCR program

- Initial Denaturation: 98°C, 30sec

30 cycles:

- Denaturation 98°C, 10s
- Anneal. X°C, 1min40s
- Extension 72°C, 2min30s

- Final extension 72°C, 10min

Gradient PCR: target 61°C, gradient 14°C

3.3 PCR template

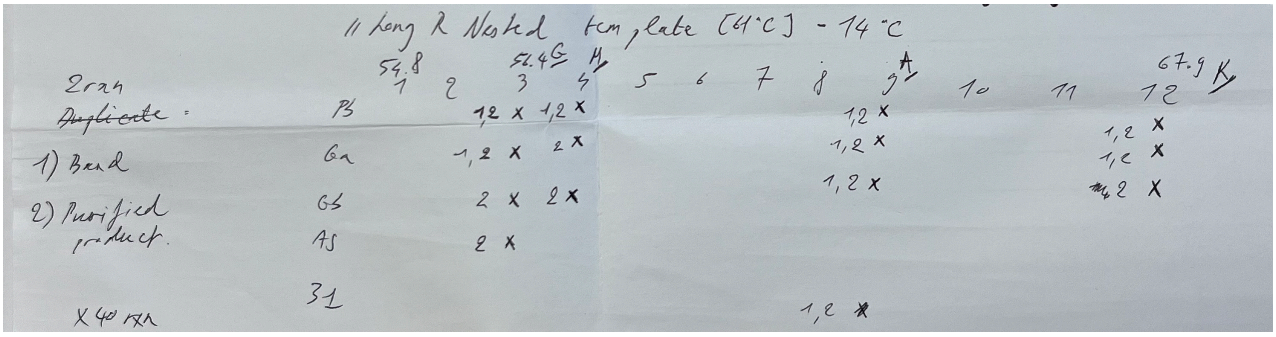

3.4 PCR Gel

All PCR product reactions were purified after PCR and some bands were also extracted (yellow crosses).

PCR gel (1.3%) made the 02/06/2022 Nanopore exp long read (nested PCR)

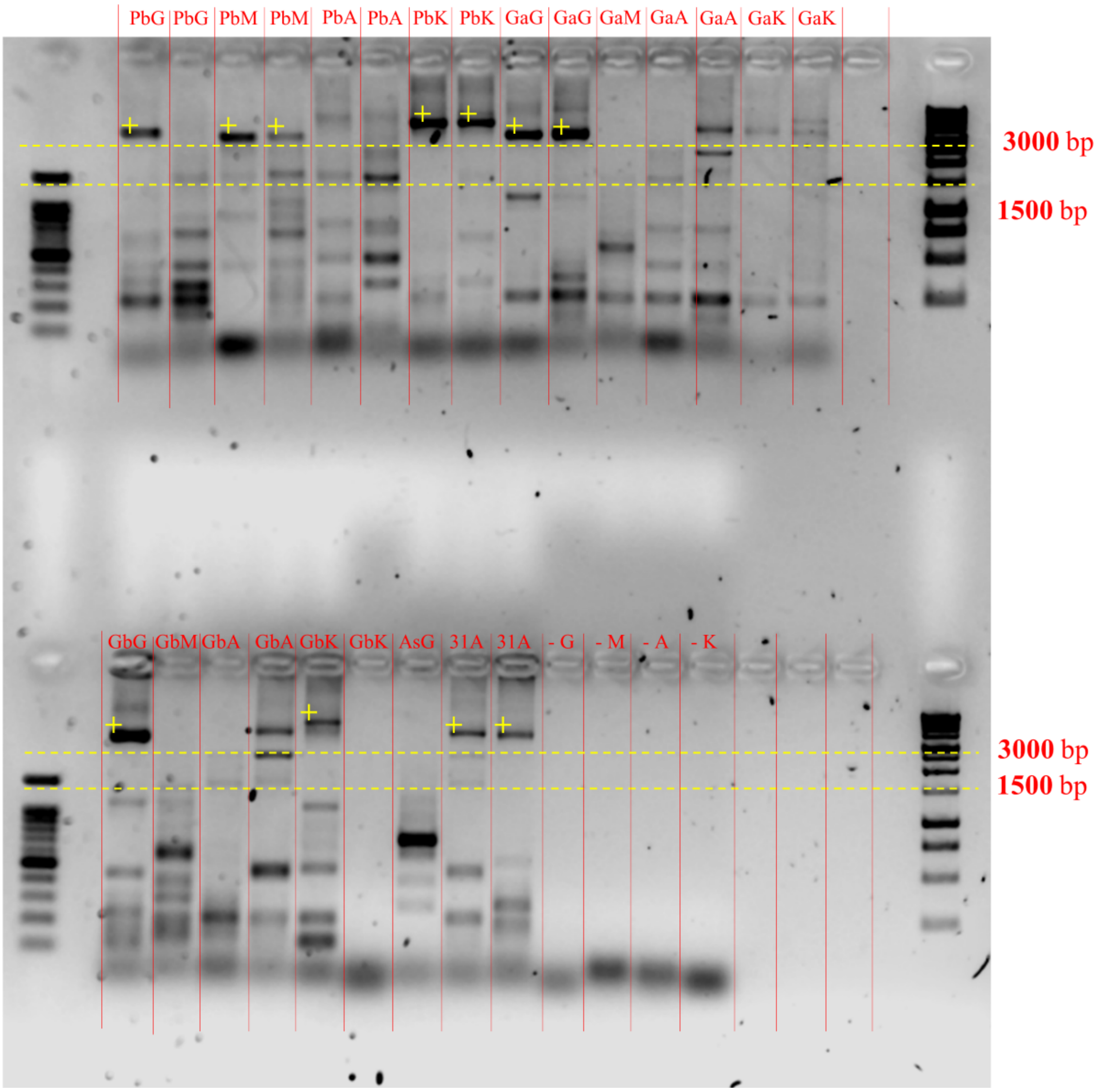

4 Nanopore Run

During this Nanopore Run, only the purified PCR products and bands from the LSU nested PCR were used for sequencing in order to obtain more long reads data:

		Pb	As	Gb	Ga	31 (A. diapernus)
	M	+				
	Greg	+		+	+	
	Api					
	Cr					
	Kinet	+			+	
	Chloro					
	Am					+
	Ichtyo					

The Nanopore library was constructed with the **native barcoding amplicons kit EXP-NBD104** and the **ligation kit SQK-LSK109**. Eventually, the following pools of amplicons and respective barcodes were used for sequencing:

Run	Sample/Pools	Initial C of pools (ng/uL)	C after End-prep step (ng/uL)	Barcode	Forward sequence	Reverse sequence	C of pooled barcode sample (ng/uL)
02/06/2022	UnonMet Pb	20.6	0.4	NB01	CACAAAGACACCGACAACTTTCTT	AAGAAAGTTGTCGGTGTCTTTGTG	0.1 (1.5 ng total in 15 µL)
02/06/2022	Pb	0.8 - 1	0.1	NB02	ACAGACGACTACAAACGGAATCGA	TCGATTCCGTTTGTAGTCGTCTGT	
02/06/2022	Gb	0.2 - 1	too low	NB03	CCTGGTAACTGGGACACAAGACTC	GAGTCTTGTGTCCCAGTTACCAGG	
02/06/2022	Ga	1.63	0.1	NB04	TAGGGAAACACGATAGAATCCGAA	TTCGGATTCTATCGTGTTTCCCTA	
02/06/2022	31	0.5	too low	NB05	AAGGTTACACAAACCCTGGACAAG	CTTGTCCAGGGTTTGTGTAACCTT	
02/06/2022	Gb spore	0.4	0.2	NB06	GACTACTTTCTGCCTTTGCGAGAA	TTCTCGCAAAGGCAGAAAGTAGTC	

Nanopore experiment report 12/05/2023

This run was different as new insects were sampled and only dissected insect tissues showing parasites under microscopic observation were retained for later PCRs. Moreover, the insect DNA pools concentrations were increased from 10 ng/µL to 100 ng/µL. Only the SSU PCR was ran at that time.

1 SSU PCR

1.1 PCR reaction

- 5x Phusion HF 5 µL
- 10 mM dNTPs 0.5 µL
- F primer 0.625 µL (20 µM)
- R primer 0.625 µL (20 µM)
- DNA 2 µL (i.e., 200 ng)
- Phusion 0.25 µL (0.5 U)
- Nuclease free water 16 µL
- Total: 23 µL

1.2 PCR program

- Initial Denaturation: 98°C, 30sec

35 cycles:

  - Denaturation 98°C, 10s
  - Anneal. X°C, 30s
  - Extension 72°C, 30s

- Final extension 72°C, 5min

1.3 PCR template

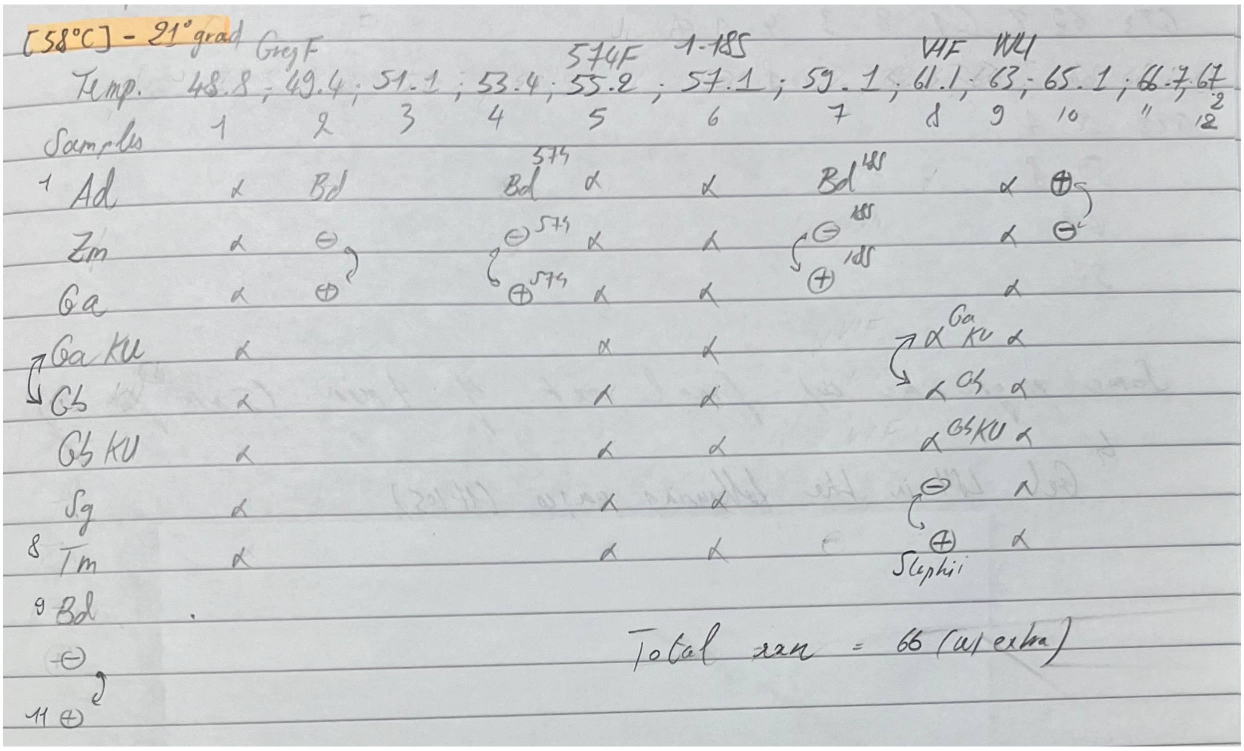

1.4 PCR Gel

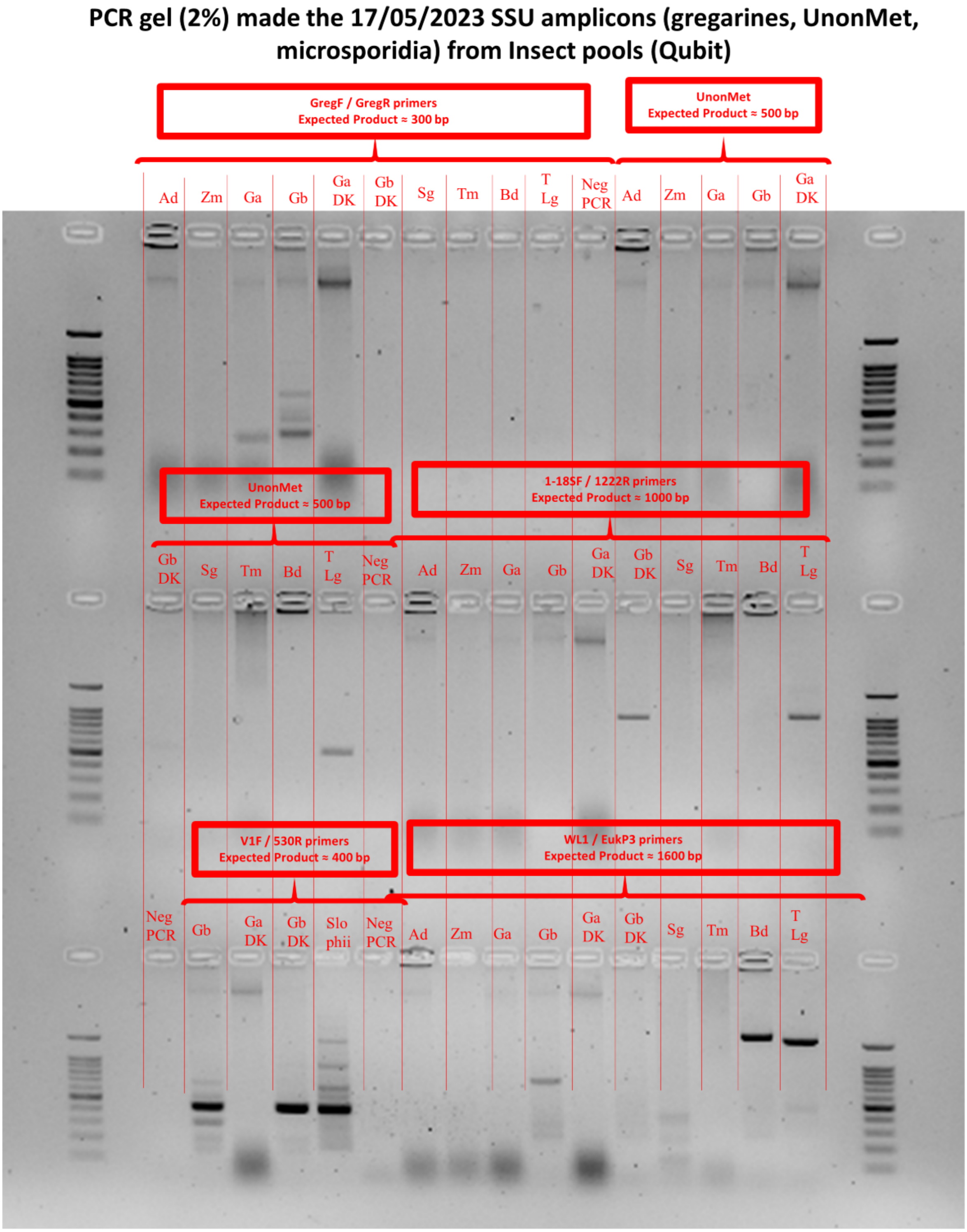

PCR gel (2%) made the 17/05/2023 SSU amplicons (gregarines, UnonMet, microsporidia) from Insect pools (Qubit)
GregF / GregR primers
Expected Product ≈ 300 bp
UnonMet
Expected Product ≈ 500 bp
Ad Zm Ga Gb Ga DK Gb DK Sg Tm Bd T Lg Neg PCR Ad Zm Ga Gb Ga DK
UnonMet
Expected Product ≈ 500 bp
1-18SF / 1222R primers
Expected Product ≈ 1000 bp
Gb DK Sg Tm Bd T Lg Neg PCR Ad Zm Ga Gb Ga DK Gb DK Sg Tm Bd T Lg
V1F / 530R primers
Expected Product ≈ 400 bp
WL1 / EukP3 primers
Expected Product ≈ 1600 bp
Neg PCR Gb Ga DK Gb DK Slo phii Neg PCR Ad Zm Ga Gb Ga DK Gb DK Sg Tm Bd T Lg

Gels with T Ga PCR products

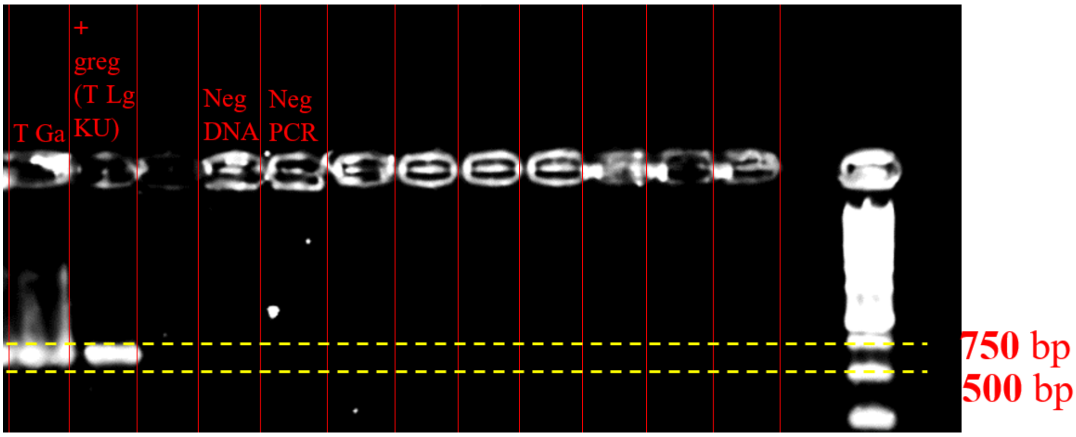

PCR gel (2%) made the 14/05/2023 SSU amplicons (gregarines, UnonMet, microsporidia) from Insect pools

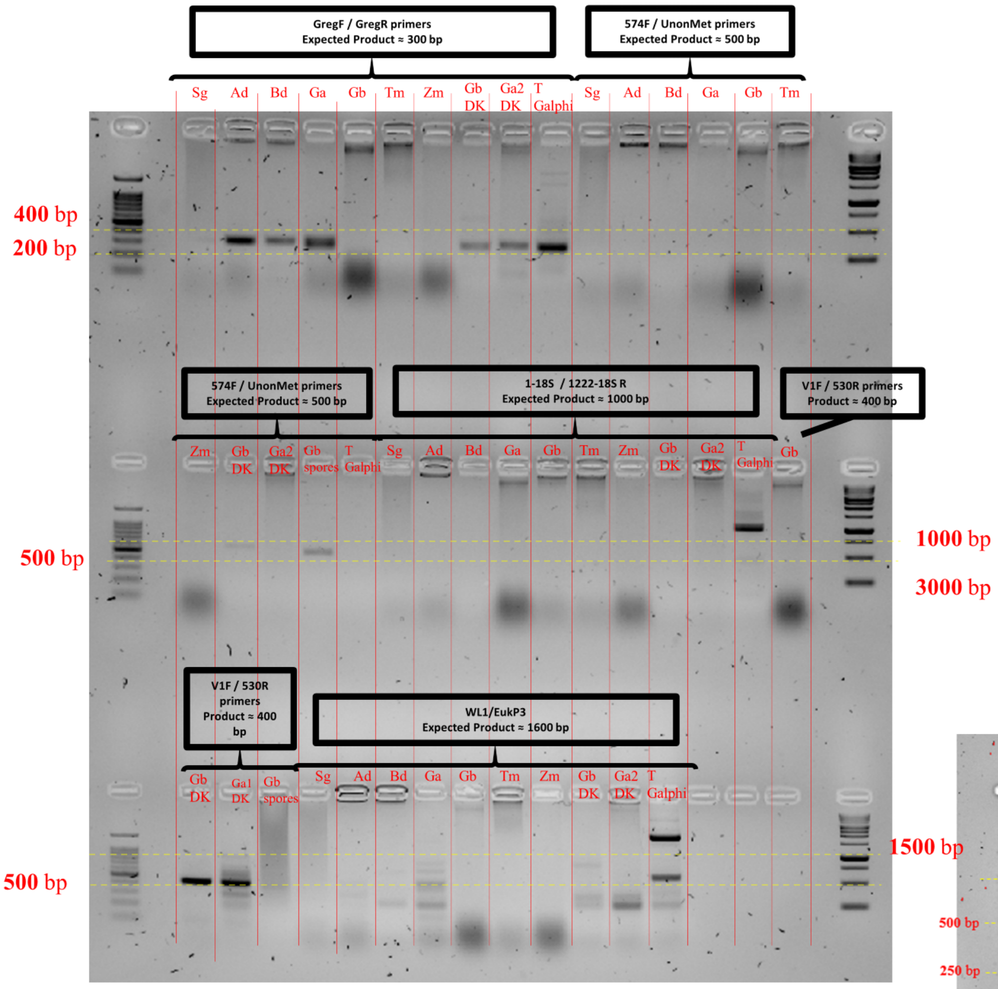

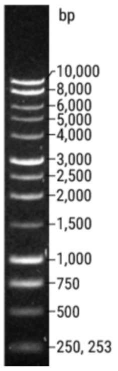

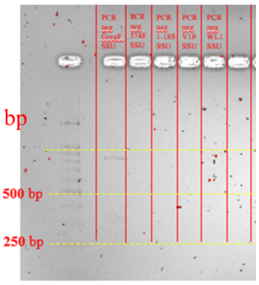

2 Nanopore Run

Purified PCR reactions were pooled equimolarly (i.e. depending on the DNA concentration and amplicon size) by insect host or parasite. The samples used to perform Nanopore sequencing were the following:

Nanopore Run	Sample/Pools	Initial C of pools (ng/uL)	C after End-prep step (ng/uL)	Barcode	Forward sequence	Reverse sequence	C of pooled barcode sample (ng/uL)
17/05/2023	T Lg	8.7	6.2	NB01	CACAAAGACACCGACAACTTTCTT	AAGAAAGTTGTCGGTGTCTTTGTG	23.6 (566 ng total in 15 µL)
17/05/2023	Bd	16.5	10.2	NB02	ACAGACGACTACAAACGGAATCGA	TCGATTCCGTTTGTAGTCGTCTGT	
17/05/2023	Tm G	9.1	7.3	NB03	CCTGGTAACTGGGACACAAGACTC	GAGTCTTGTGTCCCAGTTACCAGG	
17/05/2023	Ad G	6.12	6.5	NB04	TAGGGAAACACGATAGAATCCGAA	TTCGGATTCTATCGTGTTTCCCTA	
17/05/2023	Gb DK	8.12	23.6	NB05	AAGGTTACACAAACCCTGGACAAG	CTTGTCCAGGGTTTGTGTAACCTT	
17/05/2023	Gb	40	13.4	NB06	GACTACTTTCTGCCTTTGCGAGAA	TTCTCGCAAAGGCAGAAAGTAGTC	
17/05/2023	Ga DK / T Ga	8.06	11	NB07	AAGGATTCATTCCCACGGTAACAC	GTGTTACCGTGGGAATGAATCCTT	
17/05/2023	Ga G	15.3	4.3	NB08	ACGTAACTTGGTTTGTTCCCTGAA	TTCAGGGAACAAACCAAGTTACGT	

'T Lg' and 'T Ga' represent pure gregarine DNA from *Acheta domesticus* and *Alphitobius diaperinus* respectively. The later pool was inadvertently pooled with another insect sample and barcoded with (NB07). Reads were recovered anyway based on the expected gregarine species.

The Nanopore library was constructed with the **native barcoding amplicons kit EXP-NBD104** and the **ligation kit SQK-LSK109**.
